# Scoping review of needs for digital technology in co-creation: a health CASCADE study

**DOI:** 10.1186/s40900-025-00797-x

**Published:** 2025-10-21

**Authors:** Quentin E. A. Loisel, Qingfan An, Vinayak Anand-Kumar, George Balaskas, Homer Papadopoulos, Dimitra Pappa, Maria Giné-Garriga, Giuliana Raffaella Longworth, Sebastien F. M. Chastin

**Affiliations:** 1 School of Health and Life Science, Glasgow Caledonian University, Govan Mbeki Building, Room A258, Cowcaddens Road, Scotland, G4 0BA UK; 2 Department of Community Medicine and Rehabilitation, Umeå University, Umeå, Sweden; 3 Health Psychology and Behavioural Medicine, Constructor University, Bremen, Germany; 4 Institute of Informatics and Telecommunications, NCSR “Demokritos”, Athens, Greece; 5 Department of Digital Systems, University of Piraeus, Athens, Greece; 6 Faculty of Psychology, Education and Sport Sciences Blanquerna, Department of Sport Sciences, Universitat Ramon Llull, Barcelona, Spain; 7 Faculty of Health Sciences Blanquerna, Department of Physiotherapy, Universitat Ramon Llull, Barcelona, Spain; 8 Department of Movement and Sports Sciences, Ghent University, Ghent, Belgium

**Keywords:** Patient and public involvement (PPI), Digital technology, Co-creation, Participatory research, Participatory needs, Collective intelligence, Artificial intelligence

## Abstract

**Background:**

Patient and public involvement (PPI) is increasingly recognised as essential for meaningful, equitable, and impactful health and social care research. Co-creation is a promising involvement approach, but it faces barriers to enable its optimal potential. Digital technologies have the potential to overcome these challenges and strengthen the participatory process, but the specific technology needs that underpin effective participation and engagement remain underexplored.

**Methods:**

We conducted a comprehensive scoping review of 60 peer-reviewed studies to systematically map the digital technology needs supporting co-creation processes. Needs were extracted and thematically analysed, resulting in a structured synthesis.

**Results:**

A total of 337 distinct digital technology needs were identified and organised into five thematic areas: Ensuring Integrity, Enabling Methodology, Cognitive Needs, Group Dynamics, and Process Management. While most needs focused on functional attributes, non-functional characteristics, such as usability, scalability, and inclusivity, emerged as critical for meaningful patient and public engagement. The review highlights the fragmented articulation of technology needs across disciplines and settings. It proposes a structured framework to make latent needs visible, align stakeholder perspectives, and guide the development of digital tools. The emerging role of artificial intelligence in supporting hybrid models of involvement, along with the associated ethical challenges, is also discussed.

**Conclusion:**

This review provides the first thematic framework for understanding and addressing digital technology needs in co-creation. The findings offer a foundation for researchers, practitioners, and policy-makers within health and social care to develop and implement digital tools that enhance accessibility, engagement, and impact of participatory processes. Future research should validate and refine these insights in partnership with patients, service users, and diverse communities to ensure technological solutions foster truly inclusive and effective involvement.

**Supplementary Information:**

The online version contains supplementary material available at 10.1186/s40900-025-00797-x.

## Abstract (plain language summary)

Involving patients, carers, and the public in health and social care research (often called patient and public involvement, or PPI) helps ensure that the research addresses real needs, is more useful in practice, and remains inclusive. Today, digital technologies, such as online meetings, apps, and artificial intelligence, can help more people become involved. But what do we really need from these technologies to make everyone’s involvement easier and better?

In this study, we looked at 60 research articles to find out what kinds of digital tools are needed to support group work and co-creation in research. We discovered 337 different needs and grouped them into five main areas: making sure the process is open and equitable, so that everyone has a fair chance to contribute, supporting the methods, helping people understand and take part, working well in groups, and keeping everything organised.

We found that while many tools focus on what they do, it’s just as important that they are easy to use, work well for lots of people, and help everyone join in, especially people who may find it harder to get involved. We also discuss how new technologies like artificial intelligence can help, but why they need to be used responsibly.

Our findings can help researchers, organisations, and the public choose or design better digital tools for involving patients and the public in research. This way, everyone’s voice can be heard, and health and care research can become more inclusive and effective.

**Supplementary Information:**

The online version contains supplementary material available at 10.1186/s40900-025-00797-x.

## Introduction

Modern health and social care systems face complex and evolving challenges, ranging from global pandemics and widening health inequalities to the impacts of climate change, that have direct and profound consequences for patients, communities, and public well-being [1–4]. Responding effectively requires not only sustainable innovation but also meaningful involvement of those most affected by health and care decisions. There is now strong recognition that patient and public involvement (PPI) is essential for research and service improvement, leading to higher-quality, more relevant, and more equitable outcomes [5–7].

Within the PPI spectrum, co-creation has emerged as a promising paradigm for embedding the patient and public voice at every stage of research and innovation. It brings together patients, service users, carers, professionals, researchers, and policy-makers as equal partners. By leveraging collective intelligence and fostering shared decision-making, co-creation seeks to integrate diverse lived experiences and expertise to generate effective, widely accepted, and sustainable solutions in health and social care [8]. The participatory nature of co-creation offers a mechanism for developing more inclusive, implementable, and resilient strategies to drive systemic change [9, 10].

However, realising the full potential of co-creation is challenging in practice, and participatory approaches face significant barriers. Its iterative and collaborative nature demands versatile, resource-intensive, and highly adaptive organisational processes, which can be challenging to initiate, implement, and manage at scale [11, 12]. Furthermore, the collective intelligence and shared governance principles that underlie co-creation require careful management of risks such as groupthink [13] and power imbalances among co-creators [14], which can inhibit the integration of critical or marginalised perspectives. As the number of co-creators increases, methodological obstacles arise, such as maintaining meaningful engagement across large and diverse groups, which can limit the effectiveness of collective processes [15]. Such challenges can limit the meaningful integration of the patient and public perspective, despite policy and funding imperatives.

Digital technologies offer potential solutions to address these barriers, supporting PPI and co-creation in research and practice. From cloud computing and social media platforms to recent innovations, such as generative artificial intelligence and blockchain, these technologies enable the creation of tools that can help overcome some of these barriers, supporting the coordination, inclusivity, and adaptability required for effective co-creation processes [16, 17]. The COVID-19 pandemic starkly demonstrated the enabling role of technology for remote and distributed engagement, making participation possible when in-person involvement was restricted [18, 19]. At the same time, rapid digital innovation is transforming the ways patients and the public can contribute, collaborate, and influence research. Digital tools promise not only to enhance existing practices but also to fundamentally reshape the ways we work together, making involvement more accessible, scalable, and inclusive [20].

Yet, despite the growing emphasis on digital approaches in co-creation, a systematic understanding of the specific digital technology needs that support meaningful engagement processes remains lacking. This study aims to address this gap by systematically mapping and thematically analysing the digital technology needs that underpin effective co-creation processes. In a context where terminology and practices surrounding involvement, engagement, co-creation, and related concepts are often fragmented and overlapping across disciplines, this review adopts a deliberately broad perspective, recognising that valuable insights can be drawn from the broader field of co-creation to inform best practice in PPI and patient involvement. Our findings provide a foundation for researchers, practitioners, and policy-makers to develop and select digital tools that strengthen patient and public participation and foster more inclusive, impactful research and service improvement.

## Methods

There is a wide range of definitions of co-creation [21]; for this study, we adopted an all-encompassing definition, which incorporates any collective creativity involving a diverse set of relevant and affected actors in creative problem-solving to achieve a desired outcome [22]. For consistency, we refer to these methodologies collectively as “co-creation,” the participants as “co-creators,” and digital technologies as “technology.” For clarity, all new terms introduced in this paper are summarised with definitions in the terminology table available in Additional File 1.

Our research question was: “What are the needs for digital technology in co-creation?”. To answer this, a systematic scoping review of the literature was conducted. This study design is well-suited to answering research questions that request an overview of the literature to identify knowledge gaps and orient future research questions [23]. We followed the PRISMA guidelines for scoping reviews [24] and used artificial intelligence technologies to assist the search and selection stages. The PRISMA Checklist can be found in Additional file 2.

While no public or patient was involved, this work is part of the Health CASCADE programme [25] and serves as the foundation for developing future co-creation projects that will actively engage patients and the public. The GRIPP2-SF checklist [26] is provided in Additional file 3.

### Stage 1: Search

We examined peer-reviewed articles on co-creation processes that identified specific needs and suggested technology as a potential solution. We conducted a search using the standard Boolean format combination of keywords from the lexical field ”technology” AND “needs” AND “co-creation”:

- *Technology*: (technolog*; AI; artificial intelligence; big data; robot*; communication technolog*; ICT; information communication technolog*; deep learning; machine learning; ML; DL; IoT; internet; VR; virtual; 3D; software; computer; digital*; high tech*; cloud; voice recognition; T2S; text to speech; speech to text; NLP; natural language process*; avatar; social media; blockchain; collaboration tool; program*; API)
- *Needs*: (need; trouble; problem; issue; challenge; stake; necessity*; drive; opportunit*; limitation; solution; requirement)
- *Co-creation*: (“co-creat*” OR “co-production” OR “public participation” OR “participatory” OR “co-design” OR “user-involvement”).

We searched the following databases: Co-Creation Database (CCDB) version 1.5 (a specialised pre-existing database that comprises all peer-reviewed literature about co-creation in PubMed, ProQuest, and CINAHL from 1970 to December 2021 [22], PubMed, ProQuest, Scopus, Web of Science and CINAHL. The search within databases retrieved many records that could not be narrowed down without risking the exclusion of relevant material [22], which would have been inconsistent with the strength of the scoping review methods.

Therefore, we developed a deep-learning classification model, an artificial intelligence technology, to filter relevant material and identify relevant papers within the large pool of retrieved articles. To train this classifier, we used the human-annotated data developed during the creation of the Co-creation Database (CCDB) version 1.5 [27] as training material. Based on the BERT architecture [28], we trained an encoder-transformer model on a downstream classification task to identify documents relevant to co-creation based on their title and abstract. We employed a heuristic approach to the downstream results of the deep-learning model. The model was evaluated on unseen data, achieving an accuracy of 15% with 35% false positives and 15% false negatives.

When our classifier was ready, we conducted comprehensive Boolean searches in CCDB version 1.5, Scopus, and Web of Science from 1970 to June 2024 and in PubMed, ProQuest, and CINAHL from December 2021 to June 2024. We presented the paper’s titles and abstracts to our trained model, which classified the records. The records classified as positive were included after two rounds of deduplication: one aimed at deduplicating documents found in multiple databases and one against the papers used to produce the CCDB.

We further refined the search by using a Python script keyword-matching process. It identified records containing sentences combining at least one keyword of the set “technologies” and one of the “needs” set, which is similar to a Boolean search (e.g., [(“technolog*” OR “AI” OR “etc.”) AND (“need” OR “trouble” OR “etc.”)]), in their title or abstract. We added the condition that these terms had to appear in sentences (syntactic space terminated by a dot “.”, a question mark “?” or an exclamation mark “!”).

### Stage 2: Study selection

We used an artificial intelligence tool, ASReview, to assist with the title and abstract screening of the references obtained. Based on simulations, we could expect 95% of relevant references by screening 10% of the dataset [29]. We used the same validated process as the one used to curate the CCDB [22]. QL, SB and GB proceeded with the title and abstract AI-assisted screening. The references obtained were full-text screened by QL, GB, SC, GRL, QA, VAK, and MGG. At least two independent researchers screened each study. Any secondary source referring to a need within a paper was checked and added to the full-text screening process. Both screenings were double-blinded, and any conflicts were resolved through discussion. The selection criteria are presented in Table 1.

**Table 1:** Selection criteria

Inclusion Criteria	Exclusion Criteria
- Written in English. - Include at least one keyword of each lexical field: “technology”, “needs”, and “co-creation”. - Mention a process that adheres to our definition of co-creation. - Mention at least one need relative to this process. - Mention that technology could solve the mentioned need.	- Focus exclusively on challenges or needs without specifying that technology could solve them. - Discuss technology in the context, but not concerning addressing any specific need in the co-creation process.

### Stage 3: Charting and analysing the data

The data from the included papers was gathered through a standardised extraction table made in Excel. The extraction was organised around three sections: (1) basic bibliographic information, (2) information about the study, (3) needs and their characteristics. QL and SB piloted the table with five papers before performing the extraction. QL, GB, SC, GRL, QA, VAK, and MGG proceeded with the charting. A second researcher double-checked each extraction.

We employed thematic analysis [30] as a methodological framework to systematically categorise the diverse needs for digital technologies in co-creation. This approach was instrumental in identifying, analysing, and reporting patterns across the collected literature. One researcher (QL) read each document thoroughly to achieve deep familiarity, enabling the initial identification of relevant data points related to digital technology needs in co-creation contexts. Subsequent coding was performed iteratively; initial codes were generated to label text segments that signified distinct aspects related to digital technologies in co-creation. These codes were then examined for patterns and grouped into potential themes of digital technology needs. Each theme was rigorously reviewed and refined to ensure it captured the essence of the coded data and the overarching insights from the literature. Co-authors who undertook data charting (GB, SC, GRL, QA, VAK, MGG) reviewed a sample of codes and theme definitions for validation; any comments were reconciled through discussion until consensus was reached. The final themes and subthemes were named and defined to represent the synthesised findings accurately. Then, a narrative description of each subtheme and its overarching themes was produced.

## Results

### Search results

**Fig. 1:**
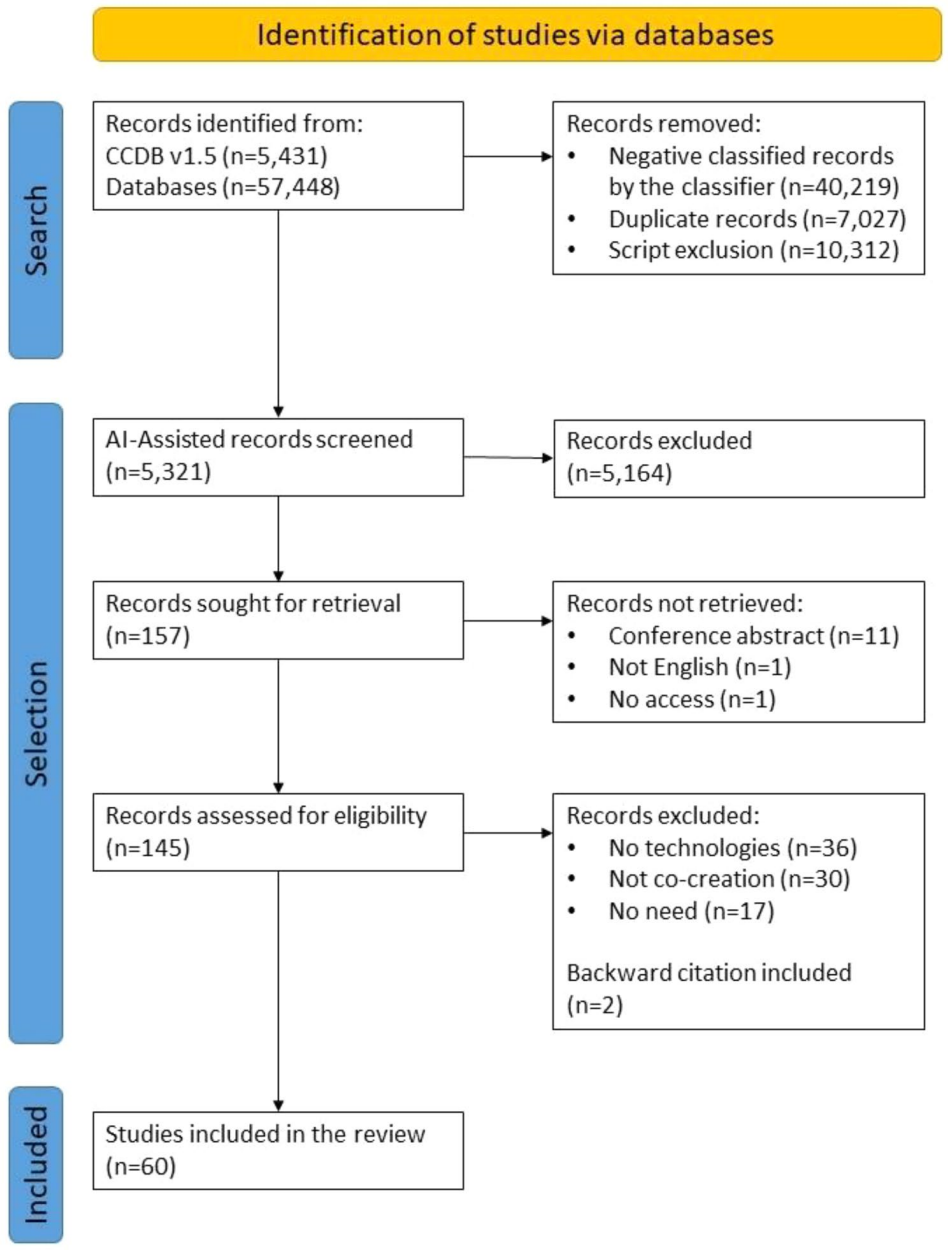
PRISMA flow chart of the selection proces

Figure 1 shows the PRISMA flow chart. The search retrieved 5,431 records from the CCDB and 57,448 from the Web of Science, Scopus, CINHAL, ProQuest, and PubMed. Deduplication and identification reduced the number of records to 5,321. After the title, abstract, and full-text screening, 60 studies were included in this review.

### Study descriptions

The dataset included a total of 60 studies. Case studies were the most common, with 19 instances (31.7%). Theoretical studies accounted for 10 entries (16.7%). Empirical studies made up six entries (10%). Five studies focused on tool or system development and evaluation (8.3%). Literature reviews were represented in 4 instances (6.7%). Framework-related studies, including theoretical frameworks, framework proposals, and framework analyses, comprised eight entries (13.3%). Finally, there were eight unique study types (13.3%) that do not fall into the previous categories, including “Randomised Trial,” “Prototype Development,” “Opinion Paper & Case Review,” and “Perspective Article.”

Regarding the field covered, Urban Planning and related topics were the most prevalent, appearing in 19 entries (31.7%). Studies in Human-Computer Interaction (HCI) appeared in seven entries (11.7%), including combinations with Participatory Design, Child-Computer Interaction, Citizen Science, and Autism Research. Environmental Sciences accounted for six entries (10%). Studies involving Public Policy and E-Government total five entries (8.3%). Studies explicitly framed within the participatory design tradition were featured in five entries (8.3%), often in connection with HCI or Urban Planning fields. Citizen Science fields appeared in three entries (5%). Public Administration was represented by three entries (5%). At the same time, Communication Design, Creative AI, Digital Democracy, Technology-Enhanced Learning, Water Management and Participatory Planning each appeared once or twice, collectively accounting for 6.7% of the dataset. Other unique areas, such as Virtual Reality & Design, Software Engineering, and Collaboration Support Systems, appeared once each, collectively making up the remaining 5% of the studies.

The goals of the studies vary widely, covering several domains of participatory design and digital co-creation. Among them, 13 studies (21.7%) aimed to explore or analyse digital tools and platforms to enhance public participation and engagement, including specific technologies such as VR, social media, WebGIS, and mobile interactions in urban design and planning. Additionally, 13 studies (21.7%) focused on developing or evaluating digital platforms or tools to support participatory processes, such as frameworks for urban co-creation, youth-centred spatial data tools, and cognitive mapping systems. A smaller subset, nine studies (15%), sought to establish frameworks and methodologies for participatory processes across various contexts, including health, environmental planning, and urban resilience. Six studies (10%) focused on integrating advanced technologies like AI, blockchain, and fog computing to address trust, security, and inclusivity in participatory design. Furthermore, seven studies (11.7%) explored the challenges and opportunities of participatory design in unique settings, including remote and culturally diverse contexts, emphasising inclusivity and collaboration. Lastly, 12 studies (20%) examined specific applications or case studies in which digital tools were applied in real-world settings, providing insights into the practical implementations of participatory technology in citizen science, climate policy, and e-governance.

The details of each study are provided in Additional file 4.

### Needs classification

**Fig. 2:**
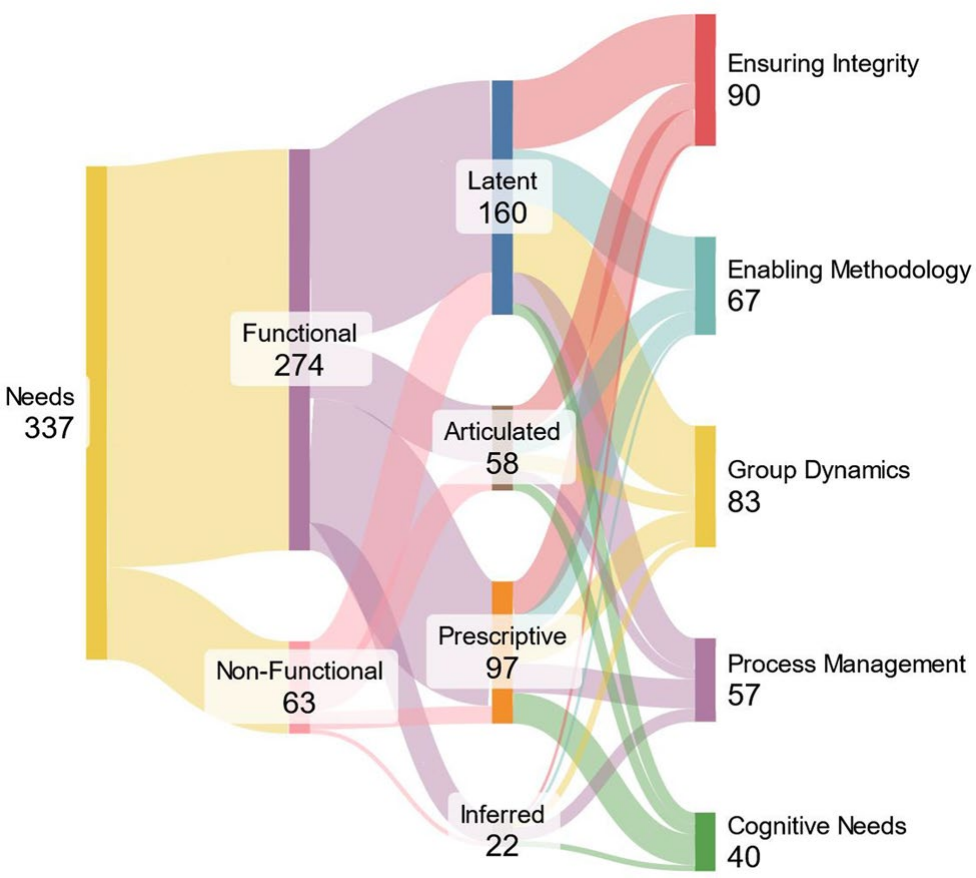
Sankey diagram for classifying the needs regarding their attribute, mode of expression and theme

The data extraction revealed 337 digital technology needs for co-creation. To highlight their singularities, they have been classified according to two dimensions: (1) their attribute and (2) their expression mode. The classification is illustrated in Fig. 2, and its details can be found in Additional file 5.

The attribute dimension concerns whether the need pertains to a technological or non-technological functional attribute. Functional attributes describe what the technology does: its core features, operations, and tasks. They are tied to the direct capabilities or behaviours that a system must exhibit to meet user requirements or business needs (e.g., “The system can authenticate users with a password” or “The application provides data export functionality”). Non-functional attributes, however, describe how well the technology performs or operates under certain conditions. They capture performance, reliability, usability, scalability, security, and maintainability. These attributes do not specify what the system should do (i.e., the functionality) but rather define the criteria or constraints for how it should do it (e.g., “The system must handle up to 10,000 simultaneous users” or “The page load time should not exceed 2 seconds”).

274 needs concern functional attributes, and 63 concern non-functional attributes. These findings reflect a common trend in requirements-gathering processes: users and stakeholders initially focus on the specific tasks and features they want a system to perform. Functional attributes are more straightforward to visualise and articulate, making them more likely to emerge. Non-functional attributes are often perceived as more abstract or technical and can be more challenging for participants to recognise or define until they encounter problems.

The second dimension identifies how the needs were expressed. *General Needs* are broad, high-level statements that identify areas for improvement without specifying particular solutions, such as “enhancing collaboration” or addressing “limited accessibility.” These needs highlight overarching goals and serve as a foundation for identifying where technological interventions might be helpful. In contrast, *Specific Needs* are detailed and actionable, specifying exact functionalities or technologies, like the requirement for a “data aggregation tool” or “GPS-based tracking.” These needs provide precise guidance for development, enabling the creation of targeted solutions. *Explicit Needs* are directly and unambiguously stated, using assertive language to indicate a straightforward requirement, such as “the system must support multilingual interfaces.” These needs leave no room for interpretation, offering clear mandates for action. On the other hand, *Implicit Needs* are indirectly suggested through context, highlighting potential areas for improvement without explicitly stating them, such as when users report that “current tools make collaboration difficult.” These needs require interpretation and contextual understanding to identify underlying requirements. Combining these characteristics allowed us to define four types of needs expression. In the case of a general and implicit need, we refer to “latent”, conveying that the need is *broad* and *underlying* or *not directly stated*. For a general and explicit need, “articulated” emphasises that the need is *broad in scope* but *clearly spelt out*. For a specific and implicit need, “inferred” suggests the need is *detailed* but must be *deduced* or *gleaned from context*. Finally, in case of a particular and explicit need, the “prescriptive” indicates the need is detailed and unambiguously stated, which, in essence, is a *clear directive*.

There are 58 articulated, 22 inferred, 160 latent and 97 prescriptive needs. These results highlight that, in co-creation contexts, many expressed needs initially emerge as broad, high-level intentions that are not fully articulated (“latent” needs). This is common when stakeholders describe overarching challenges without pinpointing a specific solution. Conversely, many “prescriptive” needs reflect when stakeholders are specific about the required functionalities or features. The smaller numbers for “articulated” and “inferred” needs suggest that while some participants can voice broad requirements, fewer needs emerge as implicit yet detailed enough to be “inferred.” This distribution shows that authors often gravitate toward general, sometimes implied, needs, with more precise directives from potentially more informed or experienced authors.

### Technological needs

**Fig. 3:**
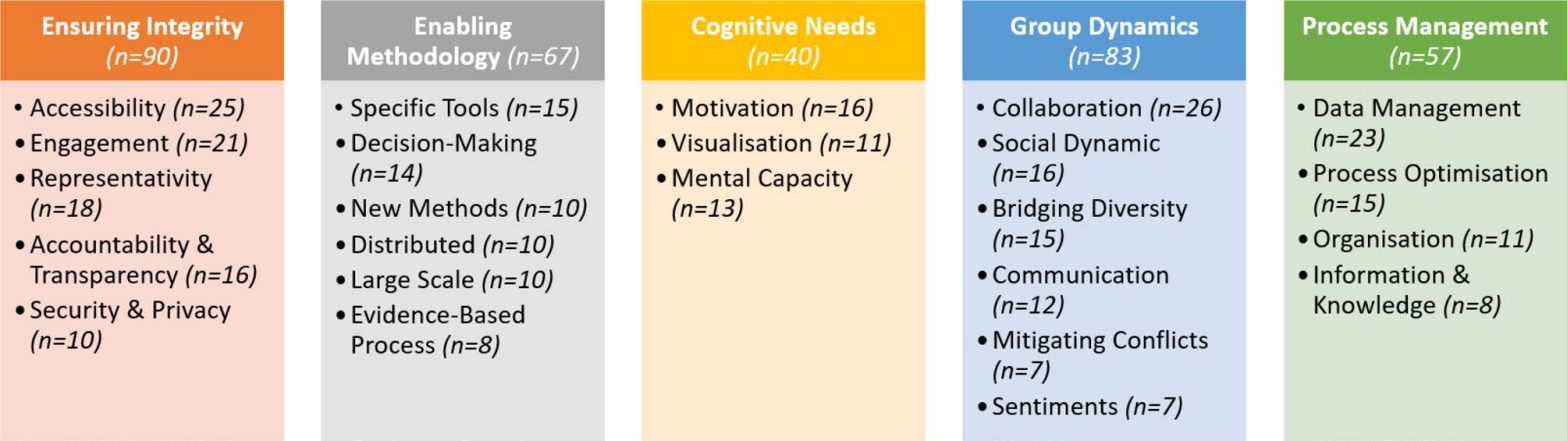
The themes and subthemes of digital technologies needed in co-creation and their corresponding quantities

The attribute and expression dimensions were considered during the thematic analysis to nuance the structuration of the five needs themes: Ensuring Integrity, Enabling Methodology, Cognitive Needs, Group Dynamics and Process Management. The themes and their 25 sub-themes are described below and illustrated in Fig. 3.

#### Ensuring integrity

90 needs and five subthemes fall into the “Ensuring Integrity” theme, reflecting technology’s need to ensure governance and co-creation values.

##### Accessibility

With 25 needs, the “Accessibility**”** subtheme focuses on designing digital tools that remove barriers to participation, ensuring that diverse and underrepresented groups can easily engage in activities through inclusive and co-creator-friendly technologies. Key priorities encompass promoting broad participation and flexible engagement by leveraging digital solutions that facilitate diverse contributions and simplify data collection [31, 32]. Ensuring inclusivity involves tailoring tools specifically for children and individuals with special needs, such as developing child-friendly platforms and neurodiversity-oriented design utilities [33, 34]. Additionally, fostering inclusion for diverse co-creator groups requires accommodating various cultural backgrounds and abilities, ensuring that interfaces are co-creator-friendly and equitable across different socioeconomic contexts [35, 36]. Addressing affordability, usability, and context sensitivity is also critical, with solutions focusing on creating cost-effective, adaptable technologies that function seamlessly in varied environments and meet specific co-creator contexts [37, 38].

##### Engagement

With 21 needs, the “Engagement**”** subtheme emphasises the development of interactive and motivating digital platforms that sustain active participation from various co-creators, mainly targeting hard-to-reach populations to maintain dynamic and ongoing collaboration. Key priorities include implementing structured approaches to attract diverse communities and ensure meaningful participation through tailored digital platforms and practical data management tools [32, 39–42]. Flexible frameworks are also essential, advocating for adaptable and centralised systems that support sustained co-creator involvement and accommodate local or domain-specific nuances [35, 37, 43–47]. Additionally, high-level directives emphasise the need for continuous engagement through long-running platforms to maintain involvement beyond the initial phases [35, 40, 48]. Furthermore, enhancing performance and co-creator experience is crucial, focusing on interactive design elements and refining collaboration practices to sustain co-creator interest and ensure high-quality interactions [49, 50].

##### Representativity

With 18 needs, the “Representativity” subtheme ensures that the co-creator pool accurately reflects the diversity of the broader community by implementing tools and strategies that minimise bias, redistribute power, and promote equitable representation. Key priorities focus on fostering inclusive representation and balancing power dynamics to ensure diverse perspectives are heard, and equitable decision-making is achieved [36, 44, 48, 51, 52]. AI-enhanced solutions are proposed to improve stakeholder inclusion by simulating varied viewpoints and aligning system designs with diverse interests, thereby bridging gaps that conventional methods may miss [53–55]. Mitigating bias in design and participation is also critical, with strategies aimed at reducing power concentration, preventing co-creator selection bias, and ensuring equitable representation [39, 55–57]. Additionally, robust data handling practices and protective measures are proposed to maintain trust and prevent manipulation or privacy breaches [36, 58].

##### Accountability and transparency

With 16 needs, the “Accountability and Transparency” subtheme involves establishing clear, open mechanisms for tracking contributions and decision-making, fostering trust among co-creators by making processes and data handling visible and understandable. Key priorities for embedding openness into digital processes include using tools that enable transparent communication, maintaining comprehensive activity logs, and ensuring fair decision-making [44, 52, 55–57]. Clear directives emphasise implementing advanced mechanisms such as blockchain-based frameworks and secure data handling practices to ensure responsibility and traceability within co-creation systems [58, 59]. Additionally, broader views position transparency and accountability as fundamental principles for building trust and legitimacy [36, 38, 55, 57, 58, 60].

##### Security and privacy

With 10 needs, the subtheme “Security and Privacy” prioritises protecting co-creator data and the integrity of collaborative environments through robust encryption, secure communication channels, and measures that safeguard against data breaches and misuse. Key priorities involve safeguarding co-creator information while maintaining transparent and trustworthy interactions. Ensuring privacy in digital collaboration requires embedding robust privacy protections and mechanisms that balance data transparency with personal anonymity, such as tracking co-creator activity without compromising identities [36, 37, 44]. Integrating robust security measures is essential, necessitating fortified digital environments, encryption, and secure network protocols to protect collaborative workspaces from vulnerabilities and unauthorised access [37, 61]. Additionally, advanced protective measures must be coupled with co-creator-friendly designs to ensure that security protocols do not hinder seamless participation [48, 59, 62].

#### Enabling methodology

67 needs and six subthemes fall into the “Enabling Methodology” theme, representing the need for technology to enable existing and new co-creation methodologies.

##### New methods

With 10 needs, the “New Methods**”** subtheme groups the needs to enable current co-creation methodologies or call for developing new ones. Key priorities involve developing participatory and geospatial tools to accomplish the optimal capacity of participatory mapping [41, 62]. Evolving digital participation frameworks are essential, focusing on integrating next-generation technology into participatory design platforms and creating adaptive, wide-ranging engagement systems supported by coherent guidelines to ensure consistency and bridge academic insights with practical applications [37, 43, 59]. Developing new online workshops and co-creation activities is crucial to enable more co-creators to contribute effectively, maintain creativity, and sustain momentum in remote settings [52, 63].

##### Distributed process

With 10 needs, the “Distributed Process**”** subtheme focuses on digital solutions that support collaboration among geographically dispersed co-creators, ensuring seamless communication and coordination in co-creation processes. Key priorities focus on facilitating remote collaboration through tools that streamline virtual participation and eliminate common friction points, supported by robust frameworks [33, 49]. Flexible and adaptive participation approaches are essential, promoting models that accommodate co-creators across various locations and time zones by integrating streamlined methods into everyday routines and simplifying interfaces [60, 64–66]. Additionally, comprehensive blueprints for organising distributed endeavours are advocated to help teams navigate complex tasks efficiently [45]. Furthermore, developing hybrid tools and inclusive virtual environments is prioritised to seamlessly blend digital and face-to-face interactions [44, 67].

##### Large scale process

With 10 needs, the “Large Scale**”** subtheme addresses the need for scalable digital tools and methodologies to accommodate extensive public engagement and participation without compromising functionality or inclusivity. Key priorities include building capacity for broad public engagement by developing digital platforms that enhance creativity and facilitate diverse contributions while organising asynchronous input from widely dispersed co-creators to maintain momentum and coordination [45, 52]. Concrete strategies emphasise the need for integrated tools to manage large volumes of responses, moderate discussions effectively, and utilise pervasive technologies for data collection and processing to derive reliable insights from extensive co-creator pools [48, 52, 56]. Additionally, ensuring scalability and inclusion is crucial, with solutions to create flexible digital methods that can accommodate expanding stakeholder groups without compromising accessibility or coherence [55, 56, 68].

##### Decision-making

With 14 needs, the “Decision-Making**”** subtheme centres on technologies that enhance and streamline the decision-making processes within co-creation, enabling more informed, transparent, and participatory outcomes. Key priorities focus on strengthening collective decision-making processes by leveraging technology that enhances informed and effective participation, such as data-driven systems for evaluating options and robust platforms that guide groups through complex choices [54, 60, 69]. Inclusive models and tools are essential to ensure adaptability to different voting contexts, simplify public input, and broaden access so that all co-creators, regardless of their familiarity with specialised software [36, 56, 57, 68, 70, 71]. Additionally, guidance and reliable systems are necessary to maintain dynamic yet well-regulated engagement, facilitate focused and transparent discussions, support deeper deliberation in complex planning scenarios, and foster diversity in collective decisions [43, 44, 68, 72].

##### Evidence-based process

With 8 needs, the “Evidence-Based Process**”** emphasises the integration of reliable data and evidence-based tools to support informed discussions, counter misinformation, and facilitate evidence-driven decision-making in co-creation. Key priorities emphasise leveraging digital technologies to enhance public discourse by filtering out inaccuracies and fostering constructive conversations grounded in reliable data [36, 71, 73]. Data-driven avenues utilise advanced functionalities such as machine learning and natural language processing to assemble, interpret, and present rigorous evidence, enabling engagement with complex findings [36, 46, 54]. Additionally, ensuring straightforward access to valuable information is needed to allow diverse audiences to form well-rounded opinions while maintaining discretion [40, 43].

##### Specific tools

With 15 needs, the “Specific Tools**”** subtheme pertains to developing and implementing specialised digital systems tailored to support participatory design, urban planning, and other co-creation activities, ensuring functionality and adaptability to co-creator needs. Key priorities include the creation of dedicated digital platforms tailored to participatory design and urban development, which integrate decision-making with collaborative communication to engage diverse co-creators effectively [74–76]. Additionally, there is a strong emphasis on developing open-ended, adaptive, and integrated systems that can evolve alongside emerging design needs and co-creator preferences, supporting a wide variety of planning contexts such as sustainability and large-scale stakeholder management while bridging domains like citizen science with participatory practices [48, 56, 77, 78]. Furthermore, customisation and empirical validation are critical, advocating for tailor-made solutions that meet specific co-creator or community demands and ensuring that digital co-creation tools are continuously refined through hands-on case studies and real-world experimentation to balance broad applicability with local adaptability [40, 73].

#### Cognitive needs

40 needs and three subthemes fall into the “Cognitive Needs” theme, encompassing the need for technologies to consider individual co-creators’ necessary mental processes, motivations, and capacities.

##### Mental capacity

With 13 needs, the “Mental Capacity**”** subtheme highlights the co-creators’ need to absorb, interpret, and apply knowledge through features designed to support learning, creativity, and cognitive ease. Key priorities include fostering creative and educational engagement by developing digital platforms that promote imaginative thinking, support open-ended innovation, and provide learning opportunities through transparent information-sharing and structured guidance [35, 66, 79]. Ensuring intuitive knowledge contribution is essential, achieved by designing simpler interfaces that allow co-creators to add spatial data and outline ideas effortlessly [48, 80, 81]. Harnessing advanced AI and structured support involves embedding intelligent functionalities that mimic human thought processes, utilising automation and machine learning to reduce manual tasks, and providing dynamic step-by-step guidance [38, 46, 63, 82, 83]. Additionally, improving clarity and reducing effort is crucial, focusing on streamlining tasks, enhancing clarity, and maintaining co-creator-friendly standards to minimise technical barriers and prevent co-creator fatigue [32, 84].

##### Motivation

With 16 needs, the “Motivation**”** subtheme focuses on the factors, incentives, and platform elements that inspire co-creators to stay committed and actively contribute throughout the co-creation process. Key priorities involve integrating playful dynamics to actively engage co-creators by incorporating game-inspired mechanisms that attract and sustain co-creator involvement, ensuring enjoyable and compelling interactions [55, 70, 72, 85]. Rewarding participation through various adaptive incentives is proposed, from token-based rewards to role-specific recognitions [36, 44, 58, 83]. Additionally, tailoring experiences through contextual and intelligent tools is promising, leveraging natural language processing and machine learning to personalise content, recommend relevant topics, and adjust roles based on co-creator needs and evolving contexts [37, 39, 44, 75, 85]. Creating an engaging digital atmosphere would further enhance motivation by designing intuitive and stimulating environments that captivate co-creators and support the interactive spirit of co-creation, ensuring that technology serves as an inviting and meaningful backdrop [51, 67].

##### Visualisation

With 11 needs, the “Visualisation” subtheme emphasises the importance of presenting data and ideas in accessible, context-driven ways that aid comprehension, inform decision-making, and maintain co-creator engagement. Key priorities involve facilitating contextual understanding and clarity by developing tools supporting data-driven discussions and presenting information in straightforward, easily interpreted visual formats, enhancing co-creator comprehension and dialogue [64, 76]. Empowering targeted applications is essential, focusing on tailored visualisation capabilities that address specific areas such as gender-responsive planning and provide robust graphical feedback to support distributed participatory design activities [31, 49, 60]. Managing and scaling complex data sets is critical, necessitating platforms that can efficiently handle extensive data volumes while offering offline visualisation capabilities to ensure continuous access to essential insights even in limited connectivity scenarios [38, 40]. Additionally, enhancing educational and cost-efficient visual engagement is needed to ensure accessibility and usability across diverse co-creator needs [35, 38, 83].

#### Group dynamics

83 needs and six subthemes fall into the “Group Dynamics” theme, illustrating the need for technology to support collective dynamics.

##### Bridging diversity

With 15 needs, the “Bridging Diversity**”** subtheme explores strategies and tools for uniting co-creators across varying cultural, generational, or disciplinary backgrounds to ensure a more representative and equitable design process. Key priorities involve fostering collaboration across generations by developing digital platforms that enable different age groups to participate inclusively, ensuring that previously overlooked voices are integrated into co-creation processes [79, 86]. Building bridges among co-creator groups is essential, emphasising improved knowledge-sharing across professional, local, and community divides, designing channels that reconcile scientific perspectives with on-the-ground realities [44, 55, 70, 75, 77]. Embracing cultural and linguistic diversity requires accommodating global co-creator bases and multicultural settings by embedding cross-linguistic capabilities, adhering to culture-specific engagement standards, and integrating translation components [37, 65, 86]. Additionally, accommodating mixed abilities and contexts is crucial, necessitating the design of flexible participation mechanisms that adapt to varying physical and cognitive skills, ensuring that co-creation spaces remain accessible and effective for all individuals [37].

##### Social dynamic

With 16 needs, the “Social Dynamic**”** subtheme focuses on how individuals and groups interact and connect during co-creation, emphasising inclusivity, empathy, and constructive engagement. Key priorities focus on encouraging empathy and counteracting hostilities by integrating features that build mutual understanding, detect and mitigate harmful language, and promote emotional sensitivity through interactive and visual means [36, 73, 87]. Strengthening collaborative ties and social connectedness is essential, potentially achieved by designing digital spaces that foster team building, support nonverbal communication, and adapt to co-creators’ emotional needs to create a strong sense of community and meaningful interactions [37, 51, 67, 72, 88]. Additionally, streamlining coordination and shared decision-making involves implementing integrated processes that seamlessly connect discussion spaces with decision-making platforms, utilising recommendation engines to highlight relevant content and facilitate consensus-building [39, 64]. An important priority is to ensure that social dynamic platforms remain research-driven, meaning that their design and use are grounded in empirical studies [64, 83].

##### Mitigating conflict

With seven needs, the “Mitigating Conflict**”** subtheme addresses mechanisms and approaches for recognising potential disagreements, preventing escalation, and transforming conflicts into productive dialogue within collaborative settings. Key priorities focus on cultivating productive dialogue by providing mechanisms that guide large, diverse groups through structured discussions and foster respectful exchanges, even on contentious issues, establishing common ground [52, 53]. Tools for conflict resolution and knowledge sharing are essential, emphasising flexible solutions that resolve clashes and integrate diverse forms of expertise into cohesive frameworks to ensure smooth and systematic group progress [35, 44, 71]. Additionally, structuring complexity requires adaptable systems capable of managing evolving problems and inherent uncertainties, along with integrated moderation and translation capabilities to overcome language, disciplinary, and contextual barriers, ensuring effective decision-making and collaboration [52, 71].

##### Understanding sentiments

With seven needs, the “Understanding Sentiments**”** subtheme highlights the importance of capturing and interpreting emotional cues so that co-creation platforms can respond adaptively to co-creators’ feelings, enhancing empathy and reducing miscommunication. Key priorities involve capturing emotional nuances in digital spaces by developing mechanisms that sense and interpret co-creators’ emotional dynamics, such as monitoring mood fluctuations and detecting nonverbal cues [37, 67]. Overcoming barriers to engagement is essential, achieved by designing welcoming digital environments that accommodate diverse emotional and communicative styles, reducing hurdles and fostering empathy-driven interactions [79, 85]. Additionally, integrating and summarising emotional data is crucial, with solutions that incorporate sentiment analysis into the co-creation process by merging affective data with other relevant information and presenting collective sentiment in meaningful ways to provide real-time feedback on group morale and engagement [52, 63].

##### Collaboration

With 26 needs, the “Collaboration**”** subtheme concentrates on creating and sustaining shared spaces, feedback mechanisms, and collective decision-making processes that enable co-creators to work effectively toward common goals. Key priorities encompass foundational solutions for multi-party collaboration by developing digital platforms that enable seamless coordination among globally distributed teams, support cross-organisational networks, and provide integrated spaces for collective planning and project development, all while ensuring robustness and efficiency at larger scales [35, 37, 52, 56, 73, 78, 79]. Streamlined approaches to feedback and annotation are essential, emphasising enhanced feedback loops and straightforward annotation tools that facilitate real-time responses and maintain organised, trustworthy input [37, 49, 61, 64, 81]. Additionally, fostering co-creative spaces and AI-supported collaboration highlights the need for environments that harness group inventiveness and problem-solving through specialised engagement spaces and AI modules that clarify system outputs and support collective brainstorming [52, 73, 82]. Facilitating consensus and structured decision-making is also crucial, with collaborative tools designed to guide teams toward agreement in complex contexts, co-creator-friendly scenario-testing modules that visualise outcomes, and structured processes that refine proposals collaboratively, promoting convergence [39, 46, 56, 66, 76].

##### Communication

With 12 needs, the “Communication**”** subtheme examines the channels, modes, and strategies that support rich and accessible information exchanges, ensuring that all co-creators can meaningfully engage in the design conversation. Key priorities involve supporting multiple modes and contexts of interaction by developing platforms that accommodate diverse communication types, including text-based exchanges and visually enriched and audio-driven interactions, while integrating advanced mechanisms to capture non-verbal signals and ensure fluid, responsive dialogues in both real-time and asynchronous settings [37, 40, 44, 51, 56, 79, 82, 83]. Tailoring communication for distributed teams is essential, necessitating tools that bridge time zones, support remote coordination, and integrate context-aware collaborative features to enhance creativity and informed decision-making throughout the project lifecycle [51, 79, 83].

#### Process management

57 needs and four subthemes fall into the “Process Management” theme, encompassing the digital support mechanisms required to coordinate co-creation.

##### Data management

With 23 needs, the “Data Management” subtheme focuses on capturing, integrating, and analysing diverse inputs, ensuring accurate, reliable, and context-rich information underpins participatory efforts. Key priorities involve geospatial data collection and integration by developing digital solutions that capture co-creator-centric geospatial insights and, for example, combine mapping with urban-planning functionalities to support comprehensive city-scale decision-making [35, 40, 43, 76, 80]. Effective data management and analytical tools are essential, emphasising the systematic organisation, filtering, and precise handling of co-creator-contributed data through advanced language-processing techniques and real-time data parsing toolkits that structure co-creator engagement [44, 46–48, 69, 73, 83]. Additionally, facilitating collective creativity and idea generation requires automated routines categorising co-creator suggestions, organising insights, analysing, and synthesising feedback [44, 46, 73, 81].

##### Information and knowledge

With eight needs, the “Information and Knowledge” subtheme addresses how project updates, feedback, and other contributions are gathered, distilled, and made accessible, enabling co-creators to turn collective insights into tangible outcomes. Key priorities involve ensuring accessible project updates and discussion summaries by establishing transparent, ongoing communication channels such as dedicated developer blogs and tools that distil lengthy discussions into clear, structured overviews, enabling all co-creators to stay informed and contribute meaningfully [64]. Evaluating and filtering diverse contributions is essential, achieved through technologies that extract the most pertinent ideas from open-ended discussions and employ sophisticated natural language processing functionalities to apply relevance scoring, enhancing clarity and facilitating efficient decision-making [63]. Additionally, advancing knowledge management requires robust information management systems that provide co-creators with seamless access to necessary materials, incorporating specialised features like semantization tools to shape knowledge bases and guide teams through co-creation best practices to ensure a consistent flow of know-how across different project phases [35, 56, 70, 77].

##### Process optimisation

With 15 needs, the “Process Optimisation” subtheme targets streamlining participatory workflows through efficient facilitation, accelerated decision-making, and reduced resource demands. Key priorities focus on streamlining participation and fostering continuous improvement by developing digital platforms that lower barriers to engagement, support straightforward input, and implement mechanisms for evaluating and refining project outcomes [40, 56, 74, 85, 89]. Automation and AI-enhanced support are crucial, emphasising the integration of natural language processing systems and AI-powered feedback engines to suggest and assess ideas, automate summarisation tasks, and generate communication aids, thereby enabling faster iterations, greater transparency, and the generation of fresh insights from co-creator inputs [63]. Additionally, holistic solutions that consolidate resources eliminate geographic and financial obstacles, minimise repetitive tasks, and create cost-efficient collaborative environments [40, 57, 65, 79, 81].

##### Organisation

With 11 needs, the “Organisation” subtheme covers the structures, platforms, and tools that align multiple co-creators, tasks, and collaborative elements within co-creation initiatives. Key priorities involve fostering engagement and recruitment by developing digital platforms that facilitate the collection of co-creator input during face-to-face sessions and strategically attract and retain co-creators through accessible, co-creator-friendly recruitment and communication channels [44, 48]. Coordinating multi-level collaboration is essential, achieved by creating platforms that align efforts across diverse teams and organisations, manage tasks seamlessly, and provide comprehensive environments for managing online engagement, including visual representations of processes to streamline the input-review cycle [40, 46, 56, 59]. Additionally, integrating real-time AI and advanced facilitation tools is crucial, with solutions that enable live interactive elements, automate facilitation tasks, and utilise machine learning algorithms to filter and categorise inputs, thereby enhancing co-creator engagement, managing complex processes, and guiding collaborative teams toward coherent outcomes [37, 46, 56, 82].

## Discussion

This review highlights 337 needs for digital technologies in co-creation. The analysis allowed us to understand and structure these needs into five categories: Ensuring Integrity, Enabling Methodology, Cognitive Needs, Group Dynamics, and Process Management. This discussion first considers the general implications of our findings for advancing co-creation practices, then contextualises each theme with existing literature, and finally explores the transformative role of artificial intelligence as a catalyst for hybrid collective intelligence in co-creation aimed at patient and public involvement (PPI) and engagement in health and social care research.

### The central role of technology in co-creation

Our results reveal a fragmented landscape of needs in the scientific literature, where functional requirements are far more prevalent than non-functional ones. Additionally, the expression of these needs varies widely, ranging from broad and implicit statements to specific and explicit directives. However, a majority of functional needs are expressed implicitly. This is not surprising since our study reviews literature from only a minority of departments specialised in technology. While this broad representation captures a realistic and heterogeneous view of co-creation practices, it also underscores the inherent complexity of systematically capturing and addressing digital needs in multi-actor ecosystems across health, social care, and other research and practice domains.

To address this gap, our study proposes a first structured organisation of these needs based on their specificity and level of articulation. By making implicit needs more visible, aligning diverse stakeholder perspectives, and translating broad technological aspirations into actionable insights, we provide a foundational framework for enhancing co-creation processes toward greater patient, public, and service user participation. Furthermore, we observed a strong synergy between the identified themes and the principles of co-creation from Chastin and colleagues [104], as illustrated in Table 2. These principles establish key conditions for authentic co-creation, and their correspondences with our themes reinforce the validity of our structure. Importantly, they illustrate that technological needs are deeply intertwined with the value-creation processes essential for co-creation to drive systemic and sustainable change involving patients and the public.

Our five-domain framework also complements and extends existing models of participatory research and co-design. For example, the MRC framework for developing and evaluating complex interventions emphasises the importance of stakeholder engagement, evidence integration, and iterative development in health research [91]. Similarly, Arnstein’s “Ladder of Participation” highlights the varying degrees of influence and power-sharing in participatory processes [92], while digital inclusion frameworks stress accessibility, equity, and usability as prerequisites for meaningful involvement [36]. Our framework adds value by systematically mapping the technological needs that underpin these participatory ideals, thereby offering a complementary perspective. Rather than proposing a new participatory model, it provides a practical lens through which researchers and practitioners can align tool development and selection with established principles of participation, co-design, and implementation science.

By aligning with established theories in co-creation (including the participatory design tradition), PPI, and engagement in health and social care, as well as other fields, our results represent a first step toward bridging the gap between high-level conceptual discussions and the practical considerations necessary for designing and implementing effective digital solutions that support co-creation and involvement in diverse research and practice settings.

**Table 2:** Correspondence between the themes and the co-creation principles

Themes	Subtheme	Corresponding Co-creation Principles
Ensuring Integrity	Accessibility	#1 (Open access to knowledge), #3 (Transparency)
	Engagement	#4 (Recognized contributions), #10 (Active participation)
	Representativity	#9 (Diversity)
	Accountability & Transparency	#2 (Legal & ethics), #3 (Transparency)
	Security & Privacy	#2 (Legal & ethics)
Enabling Methodology	Specific Tools	#7 (Evaluate), #8 (Reflect), #11 (Systematic, flexible process)
	Decision-Making	#5 (Focus on clear problem), #6 (Best evidence)
	New Methods	#8 (Reflect), #11 (Systematic, flexible process)
	Distributed	#9 (Diversity), #10 (Active participation), #11
	Large Scale	#11 (Scaling and replicability)
	Evidence-Based Process	#6 (Best evidence)
Cognitive Needs	Motivation	#10 (Active participation)
	Visualisation	#6 (Best evidence), #7 (Evaluate), #8 (Reflect)
	Mental Capacity	#10 (Active participation), #11 (Support structured, inclusive process)
Group Dynamics	Collaboration	#10 (Active participation), #11 (Systematic collaboration)
	Social Dynamic	#9 (Diversity), #10 (Active participation)
	Bridging Diversity	#9 (Diversity)
	Communication	#10 (Active participation)
	Mitigating Conflicts	#9 (Diversity), #10 (Active participation)
	Sentiments	#9 (Respect for diverse views), #10 (Support constructive engagement)
Process Management	Data Management	#2 (Legal & ethics), #3 (Transparency), #6 (Best evidence)
	Process Optimisation	#7 (Evaluate), #8 (Reflect), #11 (Enhance systematic approach)
	Organisation	#11 (Structured, replicable process)
	Information & Knowledge	#1 (Open access to knowledge), #6 (Best evidence), #9 (Diversity)

### Themes and implications

Ensuring Integrity emerged as the core theme, highlighting the critical role of technology in fostering accessibility, engagement, and balanced representation in co-creation processes. Tools must ensure diverse participation while integrating accountability mechanisms to maintain transparency in decision-making, all while addressing the inherent tension with privacy and security. For instance, accessibility-focused tools like public ICT solutions and adaptive interfaces could bridge the digital divide, enabling underrepresented groups to contribute meaningfully. These findings align with existing research emphasising accessibility and inclusivity as prerequisites for effective collaboration [93]. However, this study advances the discourse by positioning accountability as a central integrity component and addressing the challenge of balancing transparency and privacy. Technologies like blockchain offer promising solutions for transparent yet secure participation frameworks. By ensuring equity and trust, these needs form the foundation for higher-order processes, such as effective group dynamics and complex decision-making, establishing the governance structures essential for building trust and supporting advanced co-creation needs.

Enabling Methodology underscores the necessity for new technological tools and innovative methods to fully leverage technology’s potential in co-creation processes. This includes developing hybrid participation models that combine in-person and digital engagement to enhance flexibility and inclusivity. These models, alongside tools that enable large-scale, distributed, and asynchronous collaboration, break down barriers of location and time, creating opportunities for broader and more diverse co-creation. Additionally, technology must provide evidence to co-creators and support optimal decision-making approaches, ensuring that processes are both informed and effective. The findings align with prior research highlighting scalability and adaptability as essential for large-scale participatory engagements [94]. Asynchronous participation methods could enable inclusivity by allowing individuals to contribute conveniently. This study also highlights the dual focus on standardisation and customisation. Standard frameworks are proposed as a foundation tailored to balance adaptability and scalability for advancing digital co-creation processes, particularly in distributed and resource-constrained environments relevant to health and social care settings.

Cognitive Needs addresses the individual capacities of co-creators, emphasising the importance of reducing cognitive overload while sustaining engagement through intuitive, user-friendly tools. This theme focuses on overcoming the complexity of co-creation processes by motivating participants and ensuring clarity. Practical visualisation tools, gamification features, and AI-driven systems that adapt to user preferences are essential for maintaining participant focus and interest. These findings align with cognitive load theory and its application in participatory design, highlighting the importance of simplifying complex data and processes to enhance user engagement [95]. Moreover, the study emphasises the need for technologies that bridge the gap between technical experts and lay participants. Simplified interfaces, knowledge translation mechanisms, and educational resources democratise access to knowledge, enabling participants with varying expertise levels to contribute effectively. This democratisation would sustain engagement and enhance the inclusivity and quality of co-creation outcomes.

Group Dynamics fosters collaboration and resolves conflicts within diverse and distributed co-creation teams. This theme emphasises the importance of tools that bridge cultural, linguistic, and generational divides, thereby ensuring equitable participation and fostering mutual understanding in multi-stakeholder health and care settings. Culturally adaptable platforms, empathy-driven technologies, and sentiment analysis tools enable effective stakeholder communication that accommodates diverse perspectives and goals. These findings align with existing research emphasising the challenges of managing diversity in participatory processes, particularly in global initiatives where multilingual tools and culturally sensitive designs are critical for inclusivity [96]. The study also underscores the role of empathy-driven technologies, such as visualisations or augmented reality simulations, which enable participants to understand others’ perspectives, reduce misunderstandings, and foster a sense of connectedness, even in asynchronous settings. Conflict resolution tools would further enhance group dynamics by supporting structured decision-making, moderation, and bias mitigation, ensuring the equitable integration of diverse viewpoints from all stakeholder groups, including patients and the public.

Process Management is the logistical and organisational layer of needs, emphasising advanced tools to manage data, disseminate knowledge, and optimise processes. Simplifying and streamlining the process is essential to reduce its complexity and resource demands, providing much-needed support to co-creators in organising intricate workflows. The findings build on existing research highlighting the complexity of management and the complex processes of co-creation [11, 12]. Centralised digital platforms that foster collaboration and transparency by enabling resource sharing and retrieval further reduce inefficiencies. Scalability and adaptability are crucial, with modular systems designed to accommodate varying levels of complexity, from localised initiatives to global, multi-stakeholder initiatives in health and social care, aiming for systemic, sustainable change.

The choice and prioritisation of digital tools in co-creation are also influenced by the research area and methodological traditions in which they are applied. For example, studies in urban planning often emphasised geospatial and visualisation tools to support participatory mapping and city-scale decision-making [40, 62], while health-related research prioritised lived experience, accessibility, and data security as prerequisites for meaningful involvement [16, 48]. Citizen science initiatives, by contrast, frequently focused on large-scale data management and volunteer engagement systems to support distributed participation [83]. These differences reflect not only the practical demands of each field but also their underlying methodological cultures, for instance, design-driven traditions in planning, evidence-based approaches in health, and open-science practices in citizen science. Recognising these contextual influences is essential for interpreting our framework, as it underscores that digital needs are shaped by both disciplinary priorities and the participatory methodologies employed.

To illustrate its practical application, the framework can be used to guide the development of a digital platform for co-creating a community health intervention. In such a scenario, the *Ensuring Integrity* domain would help designers prioritise accessibility and privacy features to build trust and enable participation from diverse groups. *Enabling Methodology* would inform the integration of evidence-based decision-making tools and support for hybrid (online/offline) engagement. *Cognitive Needs* would guide the inclusion of user-friendly visualisation dashboards and gamified feedback to sustain motivation. *Group Dynamics* would encourage features such as multilingual communication tools and sentiment analysis to foster collaboration and empathy. Finally, *Process Management* would highlight the need for efficient data handling, knowledge sharing, and workflow coordination. Taken together, these domains offer a structured roadmap for aligning technological development with participatory values, ensuring that co-creation platforms are both functional and inclusive.

### The rise of hybrid AI-co-creators processes

The rapid evolution of artificial intelligence (AI) promises future flexible tools with applications spanning all identified themes. AI extends beyond operational enhancements to actively shape participation, collaboration, and decision-making across health and social care research and practice. Among the studies included in this review, only a limited number explicitly discussed AI-enabled co-creation. These mainly explored early-stage applications such as natural language processing for summarising contributions, AI-supported decision-making, or blockchain-AI combinations to enhance transparency and trust. While promising, such applications remain exceptions rather than the norm, underlining the emerging and experimental status of AI in co-creation.

In ensuring integrity, AI could mitigate biases to promote equitable representation, ensuring fair participation while enhancing transparency [97]. Personalised experiences like adaptive workflows and interfaces could help sustain engagement and inclusivity among patients, service users, and public contributors [98]. For enabling methodology, AI could streamline scalability and adaptability through dynamic workflows and evidence-based decision-making, transforming unstructured participant feedback into actionable insights via natural language processing (NLP) [99]. AI’s role in addressing cognitive needs could involve simplifying complex data to fit the capacity of the co-creator and sustaining motivation among participants with diverse backgrounds and levels of expertise [100]. For group dynamics, AI could mediate interactions, fostering collaboration and mitigating conflict through sentiment analysis tools that enable real-time intervention [101]. In process management, AI could automate logistical tasks, optimise resource allocation, and enhance knowledge dissemination, improving transparency and efficiency in participatory health and care research settings [102].

However, alongside these opportunities, significant challenges arise. Algorithmic bias, privacy risks, and a lack of transparency in AI systems can undermine trust, particularly in health and social care initiatives where legitimacy and inclusivity are critical [103]. These ethical challenges directly connect to the thematic framework identified in this study: for example, algorithmic bias threatens representativity under the “Ensuring Integrity” theme; lack of transparency undermines accountability and trust; and automation raises questions of autonomy and decision-making that are central to “Enabling Methodology.” Careful attention must be paid to ensure that AI-enabled solutions do not inadvertently introduce new barriers to equitable participation or reinforce existing inequalities. The stakes are proportional to the integration and responsibility of these technologies within participatory ecosystems.

Overall, AI marks a fundamental shift toward hybrid co-creation systems, where human and machine intelligence are integrated to address complex health and social challenges more inclusively, scalably, and innovatively. By leveraging AI responsibly, co-creation processes can be redefined to better support the systemic transformations required for a more inclusive and impactful future in health and social care research.

### Limitations and future research

Despite providing a comprehensive analysis for understanding digital technology needs in co-creation, this study has several fundamental limitations. Focusing on English-language literature may exclude valuable insights from other regions and cultures, and the analysis reflects researchers’ interpretations, which may not capture all perspectives. Importantly, no stakeholders or members of the public were directly involved in this study. As this was a first step designed to establish the current state of play through a systematic evidence synthesis, we followed established review procedures based solely on published literature. This allowed us to map and categorise technology needs comprehensively, and to highlight that, to date, no digital technologies directly enable patient and public involvement (PPI).

Future research should therefore actively involve stakeholders and the public to validate and expand these findings, ensuring greater practical relevance and co-created solutions. Broader inclusion of non-English literature and diverse cultural perspectives will also be essential. In addition, the evolving role of AI in supporting hybrid collective intelligence systems warrants particular attention, particularly concerning adaptive workflows and ethical considerations. Another promising direction will be to build on the observed alignment between the identified themes and established co-creation principles [104]. Future studies could explore how these principles can be operationalised through digital technologies, providing practitioners with actionable frameworks for strengthening co-creation in diverse settings. Finally, developing a comprehensive taxonomy of digital technologies in co-creation in partnership with stakeholders and communities will provide a stronger, structured framework for future development.

## Conclusion

This scoping review aimed to clarify and categorise the multifaceted digital technology needs that support co-creation processes, advancing patient and public involvement (PPI) and engagement in health and social care research and practice. By examining a broad body of literature and consolidating 337 identified needs, this study uncovers five areas to enhance co-creation: Ensuring Integrity, Enabling Methodology, Cognitive Needs, Group Dynamics, and Process Management. While functionality remains a recurring priority across these needs, the findings emphasise that non-functional requirements such as usability, scalability, inclusivity, and reliability warrant explicit and strategic attention to fully realise the transformative potential of co-creation and involvement in health and social care. Collectively, the results underline that effective co-creation hinges on the dynamic interplay between human and technological factors. To build trust, foster broad participation, and sustain long-term engagement, technologies must first secure accessibility, accountability, and privacy. From this foundation, they can evolve to support large-scale collaboration, evidence-based decision-making, and personalised, inclusive user experiences for patients, caregivers, and the public.

Moreover, emerging AI solutions present opportunities to automate logistical processes, enhance data interpretation, and facilitate richer group interactions, thereby expanding the reach and impact of public involvement in research, including patient involvement in health research, as well as broader co-creation initiatives. However, ethical considerations must remain central to AI integration to safeguard the trust and inclusivity essential for equitable and impactful health and social care innovation. Future research should refine and empirically validate these findings across diverse cultural, organisational, and geographic contexts, primarily through close partnership with patients, service users, and communities. In doing so, researchers and practitioners can contribute to building technological ecosystems that enable co-creation and involvement to their full capacity, supporting meaningful, person-centred research and improvement in health and social care.

## Supplementary Information

Below is the link to the electronic supplementary material.

Supplementary Material 1: Additional file 1, “Terminology Table”, contains a terminology table of the main terms used and introduced in the study.

Supplementary Material 2: Additional file 2, “Preferred Reporting Items for Systematic reviews and Meta-Analyses extension for Scoping Reviews (PRISMA-ScR) Checklist”, contains the PRISMA-ScR checklist guiding the scoping review process.

Supplementary Material 3: Additional file 3, “GRIPP2-SF checklist”, includes the GRIPP2-SF reporting on the patient and public involvement (PPI) dimension of this work.

Supplementary Material 4: Additional file 4, “List of Included Studies”, features a table displaying the studies included along with their type, aim, field, implied co-creation process, and associated needs.

Supplementary Material 5: Additional file 5, “List of the Needs”, presents a table detailing each identified need along with its name, type of expression, attribute, theme, and associated subtheme.

## Data Availability

The datasets supporting the conclusions are included within the article and its additional files.
