# Supplementary material for: Scoping review of needs for digital technology in co-creation: a health CASCADE study
Source: Res Involv Engagem. 2025 Oct 21;11:121. doi: 10.1186/s40900-025-00797-x (PMC12538968; doi:10.1186/s40900-025-00797-x)
Supplement: Supplementary file 1 — Supplementary Material 1: Additional file 1, “Terminology Table”, contains a terminology table of the main terms used and introduced in the study. [file 40900_2025_797_MOESM1_ESM.pdf]

# Additional file 1: Terminology Table

| Term | Definition |
|---|---|
| **Co-creation** | The umbrella concept used in this study describes collaborative processes where diverse stakeholders (patients, public, professionals, researchers, policymakers, etc.) jointly contribute to problem-solving, design, or innovation. |
| **Patient and Public Involvement (PPI)** | The application of co-creation within health research, where patients, carers, and the public are actively involved in shaping research conducted *with* or *by* them, not merely *about* them. In this paper, PPI is consistently used to denote the health-specific form of involvement. |
| **Participatory Design (PD)** | A specific methodological tradition within co-creation, originating in design and HCI fields, which emphasises structured involvement of users in the design of systems, tools, or environments. In this paper, PD is treated as a subfield of co-creation rather than a separate concept. |
| **Functional Attributes** | Features describing *what* a technology does — its core capabilities, operations, or tasks (e.g., data export, user authentication). |
| **Non-Functional Attributes** | Characteristics describing *how well* a technology performs under certain conditions (e.g., usability, scalability, inclusivity, reliability, security). |
| **Latent Needs** | Broad, underlying, and implicit needs not directly expressed (e.g., the challenge of "low participation" suggests a need for more inclusive tools). |
| **Articulated Needs** | General but explicitly stated needs, broad in scope (e.g., "the system should enhance collaboration"). |
| **Inferred Needs** | Specific but implicit needs that must be deduced from context (e.g., reports of "slow analysis" suggest a need for automated data processing). |
| **Prescriptive Needs** | Specific and explicitly stated needs, leaving no room for interpretation (e.g., "the system must support multilingual interfaces"). |
| **Ensuring Integrity (Theme)** | Technologies that secure accessibility, engagement, representativity, accountability, and privacy to maintain trust and fairness in co-creation. |
| **Enabling Methodology (Theme)** | Technologies that create, adapt, or scale participatory methods, including decision-making, evidence use, and distributed collaboration. |
| **Cognitive Needs (Theme)** | Technologies that support participants' mental processes, motivation, and ability to visualise and comprehend complex information. |
| **Group Dynamics (Theme)** | Technologies that foster collaboration, communication, conflict resolution, empathy, and inclusion across diverse groups. |
| **Process Management (Theme)** | Technologies that streamline the organisation, data handling, knowledge sharing, and optimisation of co-creation processes. |
| **Hybrid Collective Intelligence** | The integration of human and artificial intelligence to enhance decision-making, collaboration, and problem-solving in participatory processes. |
| **Transparency** | Making processes, decisions, and contributions visible and understandable to all participants, thereby fostering trust. |
| **Inclusivity** | Ensuring that participation opportunities are open, accessible, and equitable for diverse groups, including those typically underrepresented. |
