# Supplementary material for: Scoping review of needs for digital technology in co-creation: a health CASCADE study
Source: Res Involv Engagem. 2025 Oct 21;11:121. doi: 10.1186/s40900-025-00797-x (PMC12538968; doi:10.1186/s40900-025-00797-x)
Supplement: Supplementary file 3 — Supplementary Material 3: Additional file 3, “GRIPP2-SF checklist”, includes the GRIPP2-SF reporting on the patient and public involvement (PPI) dimension of this work. [file 40900_2025_797_MOESM3_ESM.pdf]

Additional file 4: List of Included Studies

Authors & Year	Need Identified	Scientific Field	Study Type	Study Aim	Co-Creation Process
Aboucaya et al., 2022	176, 195, 197, 204, 233, 242, 322	E-government, Civic Tech	Empirical Study	To analyse flaws in participatory platforms and provide recommendations for enhancing citizen engagement	Online Participatory Democracy Platform
Aikins, 2010	56, 57, 71, 226, 270, 300	Urban Planning, E-Government	Theoretical Study with Case Review	To explore how information technology (IT) can bridge theory and practice in participatory urban e-planning	E-Planning (Co-creation in Urban Planning)
Arsenopoulos et al., 2020	188, 253	Public Policy & ICT	Empirical Development and Evaluation	To develop and showcase a software-based platform that enhances public participation in decision-making, especially in addressing socio-economic and energy-related issues through crowdsourcing and open data integration.	Participatory E-Design
Barenji et al., 2021	18, 213, 288	Manufacturing & ICT	Theoretical Framework and Case Study	To propose and evaluate a blockchain and fog computing-based collaborative design and manufacturing platform to enhance customer collaboration and integrate feedback securely within the manufacturing process.	Collaborative Design & Manufacturing
Bucchetti et al., 2024	62, 113, 126, 190	Communication Design & Social Welfare	Theoretical Framework with Case Study	To explore communication design strategies for remote participatory processes, particularly for enhancing human interactions and inclusivity in virtual settings.	Remote Co-Design
Chen, 2021	123	Virtual Reality & Design	Theoretical Framework and Case Study	To explore the use of Virtual Reality and telepresence systems in co-design research to enhance collaborative creation and user immersion.	Hybrid Co-Design
Constantin and Hourcade, 2018	247	Human-Computer Interaction & Autism Research	Empirical Development and Evaluation	To develop a technology-based tool to facilitate brainstorming in participatory design (PD) sessions with children with Autism Spectrum Disorders (ASD), helping to reduce anxiety and enhance creativity.	Participatory Design with ASD Focus
Constantin et al., 2021	2, 27, 72, 88, 89, 116, 118, 127, 128, 162, 167, 198, 241, 248, 249, 271, 272, 289, 290, 311, 320	Human-Computer Interaction & Child-Computer Interaction	Opinion Paper & Case Review	To explore distributed participatory design (DPD) with children and to address the unique challenges and opportunities presented by remote participation, especially during the COVID-19 pandemic.	Distributed Participatory Design (DPD) with Children
Dalsgaard et al., 2022	3, 13, 63, 207, 250	Urban Development & Public Institutions	Case Study Analysis	To examine the challenges of participatory design in large-scale public projects, particularly within the development of a multimedia public library.	Participatory Design

Du et al., 2024	100, 183, 306	Urban Planning & AI	Systematic Literature Review	To evaluate the strengths, weaknesses, opportunities, and threats (SWOT) of integrating AI in participatory planning for urban development and to guide future improvements.	AI-Enabled Participatory Planning
Dufendach et al., 2017	193	Healthcare	Randomised Trial	To reduce user effort in co-design of a software user interface by developing a web-based platform	Participatory Design
Eilola et al., 2021	177, 194	Land Use and Environmental Planning	Case Study	To examine the benefits and limitations of participatory mapping using high-resolution remote sensing images in Tanzania	Participatory Mapping
Fessler et al., 2024	4, 168, 329	Urban Planning & Gender Studies	Case Study	To explore the use of digital tools in promoting gender-responsive public spaces	Digitally Supported Participation
Fredericks and Foth, 2013	5, 175, 184	Urban Planning	Case Study	To explore the role of social media and web 2.0 tools in enhancing public participation in planning	Augmented Public Participation
García-Holgado et al., 2020	161, 227	Citizen Science & Digital Society	Framework Analysis with Case Study	To analyse the WYRED framework for engaging young people in citizen science through a technological ecosystem	Technologically Enhanced Citizen Science
Giesen and Süpke, 2011	19, 91, 234	Sustainable Project Planning & Environmental Engineering	System Development with Case Study	To develop and evaluate the ProPlaNET system, a collaborative tool for sustainable project planning that incorporates participatory decision-making	Collaborative Planning
Heintz and Law, 2018	102, 141, 190, 228, 230, 236, 254	Human-Computer Interaction (HCI) & Participatory Design	Tool Development and Evaluation	To develop and evaluate PDotCapturer and PDotAnalyser, tools supporting asynchronous idea capturing and analysis in participatory design	Asynchronous Participatory Design
Heintz et al., 2014	44, 181, 225, 330	Human-Computer Interaction (HCI) & Distributed Participatory Design (DPD)	Tool Development and Preliminary Evaluation	To develop and evaluate Pdot, a tool for supporting distributed participatory design with online annotation capabilities	Distributed Participatory Design
Helbing et al., 2023	6, 38, 103, 104, 144, 232, 240, 291, 307, 312	Digital Democracy & Computational Social Science	Theoretical Framework with Case Studies	To explore how digital tools can support democratic processes, enhance civic engagement, and improve decision-making and transparency	Digitally Assisted Democracy
Hennig et al., 2023	166, 229	Geospatial Technologies & Citizen Science	Case Study	To create a youth-centred data collection tool for contributing spatial data on urban green areas to promote child- and youth-friendly urban development.	User-Centered Contributory Citizen Science
Hess et al., 2013	45, 129, 212, 231, 235, 273, 274, 323	Human-Computer Interaction & Participatory Design	Case Study	To explore participatory product development with online communities using social technologies, highlighting challenges in heterogeneity, role management, and process structuring.	Distributed Participatory Design
Horlitz, 2007	14, 39, 281, 313	Water Management & Participatory Planning	Theoretical Framework and Case Analysis	To explore the role of model interfaces in supporting participatory water management and improving public engagement through accessible and interactive tools	Interactive Public Participation
Jannack et al., 2015	60, 61, 105, 145, 163, 174, 199, 203,	Urban Planning & Participatory Design	Theoretical Framework with Case Study	To present a blueprint for a collaborative urban design environment that enables large-scale	Massive-Scale Collaborative Urban Design

	224, 262, 263, 286, 317			public engagement through a structured digital co-design platform	
Joubert and Wishart, 2012	73	Technology Enhanced Learning & Education	Comparative Case Study	To analyse lessons learned from two initiatives using digital technologies to facilitate knowledge building among school students and researchers in participatory contexts	Computer-Mediated Collaboration
Jutraz and Zupancic, 2012 [1]	46, 164, 196, 208	Urban Design	Empirical Study	To explore digital tools that enhance public participation in urban design through 3D ICC	Co-Design
Jutraz and Zupancic, 2012 [2]	28, 73, 92, 169, 209, 214, 326	Urban Design	Theoretical Study	To identify and define digital tools and criteria for effective cooperative urban design	Cooperative Design
Katapally, 2019	17, 30, 93, 106, 142, 255, 266, 292, 318	Public Health, Technology	Theoretical Framework	To establish a framework for integrating citizen science with participatory research in health contexts	Citizen Science, Participatory Research
Kelley and Johnston, 2012	64, 119, 134, 202	Public Administration, Game Studies	Theoretical Framework	To explore how serious games can enhance public engagement and open governance platforms	Open Governance, Serious Games
Krishnamurthy et al., 2013	130, 218	Public Policy, Information Systems	Theoretical Framework	To explore the role of information systems in generating empathy within participatory governance platforms	Participatory Platforms
Leonardi et al., 2023	7, 20, 21, 42, 68, 94, 95, 107, 135, 143, 146, 245, 285, 319, 321, 324	Public Administration	Case Study	To explore the opportunities and barriers in using ICT to support cross-organizational co-design in public administrations	Co-Design
Lieven et al., 2021	8, 51, 52, 121, 136, 275, 276, 282, 287, 303	Urban Planning and Development	Case Study	To explore the integration of digital tools in urban co-creation processes, focusing on participation technologies such as digital touch tables and AI-based feedback mechanisms.	Co-Creation
Luusua et al., 2023	9, 47, 109, 110, 138	Participatory Design, Remote Collaboration	Theoretical Exploration	To explore and theorise hybrid, multi-site participatory design (HMPD) methods for culturally diverse global contexts	Hybrid Multi-site Participatory Design
Mačiulienė and Skaržauskienė, 2016	22, 131, 251, 301, 314	Business Research	Empirical Research	To assess the co-creation capabilities of networked collaboration platforms using social indices	Networked Collaboration Platforms
Mahmoud and Arima, 2011	48, 147, 189, 331	Urban Planning	Case Study	To develop and evaluate a web-based Decision Support System (DSS) for enhanced public participation in decision-making related to urban planning.	Public Participation
Mariani et al., 2023	43, 108, 139, 140, 153, 154	Public Sector Innovation	Literature Review	To explore how design thinking methodologies can reinforce e-participation for improved digital public services.	Co-Creation
Matsumoto et al., 2024	81, 278	Human-Computer Interaction	Case Study	To explore how purpose formation can be facilitated among multiple stakeholders through AI-supported dialogue.	Co-Creation
McCormack et al., 2020	86, 148, 219, 246, 295	Creative AI and Design	Theoretical Analysis	To identify and discuss design considerations for real-time,	Co-Creation

				collaborative AI systems in creative settings.	
Mougiakou et al., 2023	50, 293	Urban Planning and Environmental Science	Case Study	To develop and apply a methodological framework for participatory spatial planning using WebGIS tools, particularly for urban areas with unique challenges.	Public Participation
Münster et al., 2017	1, 15, 29, 69, 155, 156, 205, 279, 280, 315, 316	Urban Planning & Digital Tools	Literature Review & Case Studies	To examine how digital tools can enhance public participation in urban design and identify key challenges and promising methods for massive-scale participation.	Public Participation
Panagiotopoulou and Stratigea, 2017	31, 96, 173, 178, 215, 216, 267, 268, 277, 308, 325, 332, 333	Urban Planning & ICT	Literature Review	To explore the use of spatial data management and visualization tools in enhancing participatory e-planning within smart city contexts.	Public Participation
Pejovic and Skarlatidou, 2020	157, 170, 296, 334, 335	Human-Computer Interaction & Citizen Science	Empirical Research	To explore design challenges in mobile interaction for extreme citizen science, focusing on usability issues in rural, developing regions.	Extreme Citizen Science
Pipan, 2018	23, 101, 182, 210	Urban Planning	Comparative Study	To explore how interactive tangible planning support systems (PSSs) can enhance access to non-professionals' spatial data and support stakeholder consensus-making.	Co-Creation
Recalde et al., 2020	24	Urban Planning	Prototype Development	To develop a cognitive urban planning platform prototype that integrates citizen collaboration to improve urban resilience.	Co-Creation
Reith et al., 2021	112, 124, 132, 133, 201, 252	Landscape Architecture	Case Study (Remote)	To explore remote participatory design tools and methods for enhancing engagement and collaboration in schoolyard co-design.	Co-Design
Reynante et al., 2021	16, 34, 65, 66, 74, 75, 76, 77, 82, 114, 115, 117, 158, 221, 238, 239, 260, 297, 302	Civic Design	Theoretical Framework	To develop a framework that integrates public participation, crowdsourcing, and design thinking to address large-scale civic issues.	Open Civic Design
Roszczyńska-Kurasińska and Wróblewska, 2023	25, 26, 35, 36, 58, 87, 97, 137, 159, 222, 258	Environmental Participation	Explorative Study	To examine the technological solutions supporting citizen environmental participation in Poland, with a focus on grassroots movements	Citizen Science
Shaikh et al., 2023	53, 54, 55, 149, 294, 327	E-Governance	System Proposal	To propose a blockchain-based e-participation system that enhances democratic engagement by ensuring security, transparency, and decentralised citizen participation.	E-Participation
Siemon et al., 2019	152	Collaboration Support Systems	Framework Proposal	To develop a framework for collaboration support systems that enhance team performance by addressing cognitive and social factors.	Collaborative Teams

Skarlatidou et al., 2019	32, 67, 125, 160, 256, 257, 261, 305, 336	Environmental Citizen Science	Systematic Literature Review	To identify user needs and best practices for digital technologies in citizen science, focusing on volunteer engagement and usability.	Citizen Science
Skarzauskiene et al., 2023	37, 78	Environmental Science	Case Study	To explore the role of citizen science within climate assemblies and how it can foster co-creation and engagement in climate policy development.	Citizen Science
Slingerland et al., 2022	49, 70, 79	Urban Planning/Place-Making	Case Study	To explore challenges and opportunities of distributed participatory design in place-making activities focusing on the impacts of digital transformation due to COVID-19.	Participatory Design
Stelzle et al., 2017	10, 40, 90, 298	Urban Development	Case Study	To explore decision-making processes in digital participatory urban design and translate them into effective digital tools and methods	Co-Design, Co-Decision
Šuklje Erjavec and Ruchinskaya , 2019	11, 80, 83, 165, 171, 200, 223, 269, 283, 337	Urban Planning	Case Study	To explore how co-creation and inclusiveness can be enhanced in public open spaces and the role of digital tools in facilitating these aspects.	Co-Creation
van Kouwen et al., 2009	41, 59, 84, 85	Environmental Management	Empirical Study	To explore computer-supported cognitive mapping for structuring complex participatory problems	Participatory Problem Structuring
Walsh and Foss, 2015	33, 120, 172, 185, 186, 187, 220, 237, 243, 259	Child-Computer Interaction	Empirical Research	To develop and evaluate an online environment for distributed, intergenerational co-design involving children as design partners across locations	Distributed Intergenerational Co-Design
Whelan, 2024	206, 217, 310, 328	Participatory Design	Theoretical Study	To explore how blockchain can enhance participatory design by addressing issues related to trust, power dynamics, and participation	Participatory Design
Winschiers-Theophilus et al., 2022	12, 191, 192	Child-Computer Interaction	Empirical Research	To explore distributed co-design with children across diverse geographical and cultural contexts, promoting a transcultural approach	Distributed Co-Design
Wong et al., 2016	122, 179	Information Systems	Theoretical and Framework Development	To examine how mobile technologies can support innovation co-creation processes and provide a roadmap for developing mobile ecosystems	Innovation Co-Creation
Zellner, 2024	99, 150, 151, 244, 264, 265, 299, 304, 300	Environmental Planning	Perspective Article	To explore the potential of participatory modelling in collaborative landscape and environmental planning for addressing complex issues	Participatory Modeling
Zhu et al., 2011	98, 111, 211, 284	Software Engineering	Theoretical and Framework Development	To propose the Hive-Mind Space (HMS) model to support creative distributed collaborative design by addressing communication gaps among diverse stakeholders	Distributed Collaborative Design
