# Supplementary material for: Scoping review of needs for digital technology in co-creation: a health CASCADE study
Source: Res Involv Engagem. 2025 Oct 21;11:121. doi: 10.1186/s40900-025-00797-x (PMC12538968; doi:10.1186/s40900-025-00797-x)
Supplement: Supplementary file 4 — Supplementary Material 4: Additional file 4, “List of Included Studies”, features a table displaying the studies included along with their type, aim, field, implied co-creation process, and associated needs. [file 40900_2025_797_MOESM4_ESM.pdf]

Additional file 3: GRIPP2-SF checklist

Section / Item	Response
**Aim**	The aim of this scoping review was to systematically map and synthesise the digital technology needs that underpin effective co-creation processes involving patients and the public, to inform and advance meaningful patient and public involvement (PPI) practices in health and social care research.
**Methods**	While no patients or members of the public were directly involved in designing, conducting, or authoring this review, all included studies addressed co-creation processes where patients, service users, or members of the public were active contributors to research and innovation. The review extracted and analysed technology needs supporting participation and engagement in these contexts.
**Study results (Outcomes)**	None of the included studies reported formal PPI as defined by GRIPP2 (e.g., public or patients as co-researchers or co-authors). However, all studies described co-creation or participatory methods in which patients, citizens, or the public played central, active roles. The synthesis identified 337 distinct digital technology needs relevant for enhancing the accessibility, engagement, and impact of participatory processes.
**Discussion/Conclusions**	This review advances understanding of how digital technologies can support PPI by providing a structured thematic framework of technology needs for co-creation. While formal PPI reporting remains limited, the findings highlight both the breadth of public involvement in practice and the importance of non-functional technology characteristics (e.g., usability, inclusivity) for meaningful engagement. The review recommends further research in partnership with patients and the public to ensure the development of digital tools that enable truly inclusive and practical involvement.
**Reflections/Critical perspective**	Although this review did not incorporate direct PPI in its process, its exclusive focus on studies involving public and patient participation ensures strong relevance to PPI stakeholders. Future reviews and technology development should consider these findings, with direct involvement of patients, service users, and diverse communities, to enrich research outcomes and model best practices in patient and public involvement (PPI).
