# Supplementary material for: Scoping review of needs for digital technology in co-creation: a health CASCADE study
Source: Res Involv Engagem. 2025 Oct 21;11:121. doi: 10.1186/s40900-025-00797-x (PMC12538968; doi:10.1186/s40900-025-00797-x)
Supplement: Supplementary file 5 — Supplementary Material 5: Additional file 5, “List of the Needs”, presents a table detailing each identified need along with its name, type of expression, attribute, theme, and associated subtheme. [file 40900_2025_797_MOESM5_ESM.pdf]

Additional file 5: List of the Needs

Theme	Sub-theme	#	Need Name	Expression	Attribute
Cognitive Needs	Mental Capacity	17	Tools for Knowledge Translation	Latent	Functional
Cognitive Needs	Mental Capacity	184	Improved Accessibility and Clarity of Information	Articulated	Non-Functional
Cognitive Needs	Mental Capacity	187	Creative Expression Tools for Distributed Co-Design	Articulated	Functional
Cognitive Needs	Mental Capacity	193	Effort Reduction in Co-Design Participation	Articulated	Non-Functional
Cognitive Needs	Mental Capacity	196	Informative and Educational Tools for Public Participation	Articulated	Functional
Cognitive Needs	Mental Capacity	200	Educational Tools for Skill and Knowledge Sharing	Articulated	Functional
Cognitive Needs	Mental Capacity	229	Simplified and Intuitive Spatial Data Contribution Applications	Inferred	Functional
Cognitive Needs	Mental Capacity	230	Flexible Expression Tools in Idea Specification	Inferred	Functional
Cognitive Needs	Mental Capacity	295	AI with Human Mental Modelling	Prescriptive	Functional
Cognitive Needs	Mental Capacity	296	Intuitive Knowledge Organisation	Prescriptive	Functional
Cognitive Needs	Mental Capacity	303	Automated Title and Text Differentiation	Prescriptive	Functional
Cognitive Needs	Mental Capacity	304	ML-Driven Support Tools for Skill Development and Engagement	Prescriptive	Functional
Cognitive Needs	Mental Capacity	305	Multi-Format Tutorial Resources	Prescriptive	Functional
Cognitive Needs	Motivation	62	Interactive and Stimulating Virtual Environments	Latent	Non-Functional
Cognitive Needs	Motivation	63	Context-Integrated Participation Features	Latent	Functional
Cognitive Needs	Motivation	64	Motivation Enhancement Tools for Co-Creators	Latent	Functional
Cognitive Needs	Motivation	65	Diverse Incentive Mechanisms for Participant Engagement	Latent	Functional
Cognitive Needs	Motivation	66	Adaptive Role Assignment for Participation Platforms	Latent	Functional
Cognitive Needs	Motivation	67	Volunteer Retention Support Features	Latent	Functional
Cognitive Needs	Motivation	201	Engaging and Meaningful Technology for Participant Involvement	Articulated	Non-Functional
Cognitive Needs	Motivation	202	Enhanced Gamification Strategies for Engagement	Articulated	Functional
Cognitive Needs	Motivation	233	Topic Recommendation System Using NLP	Inferred	Functional
Cognitive Needs	Motivation	310	Tokenized Incentives for Active Participation	Prescriptive	Functional
Cognitive Needs	Motivation	311	ML-Powered Interest Tools with Crowdsourcing Features	Prescriptive	Functional

Cognitive Needs	Motivation	312	Incentive Systems for Effective Information Gathering	Prescriptive	Functional
Cognitive Needs	Motivation	313	Engaging Online Platforms for Interactive e-Participation	Prescriptive	Functional
Cognitive Needs	Motivation	314	Advanced Gamification Features	Prescriptive	Functional
Cognitive Needs	Motivation	315	Gamification for Enhanced Motivation	Prescriptive	Functional
Cognitive Needs	Motivation	316	Sustained Engagement through Gamification	Prescriptive	Functional
Cognitive Needs	Visualisation	101	Simplified Data Visualization for Non-Specialists	Latent	Functional
Cognitive Needs	Visualisation	235	Visualization Tools for Context-Focused Discussion	Inferred	Functional
Cognitive Needs	Visualisation	329	Enhanced Data Collection and Visualization for Gender-Responsive Planning	Prescriptive	Functional
Cognitive Needs	Visualisation	330	Support for Graphical Feedback in Distributed PD Tools	Prescriptive	Functional
Cognitive Needs	Visualisation	331	Enhanced Visualization Tools	Prescriptive	Functional
Cognitive Needs	Visualisation	332	Large-Scale Spatial Data Visualization	Prescriptive	Functional
Cognitive Needs	Visualisation	333	Management and Visualition of Large Data Set	Prescriptive	Functional
Cognitive Needs	Visualisation	334	Accessible Data Representation	Prescriptive	Non-Functional
Cognitive Needs	Visualisation	335	Offline Data Visualization Capability	Prescriptive	Functional
Cognitive Needs	Visualisation	336	Visual Aids in Educational Content	Prescriptive	Functional
Cognitive Needs	Visualisation	337	Cost-Effective Engagement and Visualization Tools	Prescriptive	Functional
Group Dynamics	Collaboration	18	Efficient and Trustworthy Customer Feedback Integration	Latent	Functional
Group Dynamics	Collaboration	19	Widespread Integrated Collaborative Planning Tools	Latent	Functional
Group Dynamics	Collaboration	20	Problem-Solving and Consensus-Building Tools	Latent	Functional
Group Dynamics	Collaboration	21	Cross-Organizational Collaborative Platforms	Latent	Functional
Group Dynamics	Collaboration	22	Collaboration Organization Tools for Diverse Actor Networks	Latent	Functional
Group Dynamics	Collaboration	23	Tools for Facilitating Stakeholder Consensus	Latent	Functional
Group Dynamics	Collaboration	24	Technology to Enable Collaboration	Latent	Functional
Group Dynamics	Collaboration	25	Networking Tools for Cross-Organizational Collaboration	Latent	Functional
Group Dynamics	Collaboration	26	Basic Collaborative Tools for Enhanced Non-Profit Efficiency	Latent	Functional
Group Dynamics	Collaboration	86	Enhancing Collaborative Creativity	Latent	Functional

Group Dynamics	Collaboration	87	Collaborative Tools for Defining Research Questions	Latent	Functional
Group Dynamics	Collaboration	162	Asynchronous Tools for Global Collaboration	Articulated	Functional
Group Dynamics	Collaboration	163	Collaborative Platforms for Shared Project Development	Articulated	Functional
Group Dynamics	Collaboration	164	Holistic Platform for Communication and Collaboration in Design	Articulated	Functional
Group Dynamics	Collaboration	165	Scalable and Efficient Digital Collaboration Tools	Articulated	Non-Functional
Group Dynamics	Collaboration	174	Collaborative Creative Space for Public Engagement	Articulated	Functional
Group Dynamics	Collaboration	180	Enhanced Feedback Integration in Participatory Design Tools	Articulated	Functional
Group Dynamics	Collaboration	181	Powerful and User-Friendly Annotation Tool	Articulated	Functional
Group Dynamics	Collaboration	219	AI Explanation and Justification	Inferred	Functional
Group Dynamics	Collaboration	242	Collaborative Editing for Proposal Development	Prescriptive	Functional
Group Dynamics	Collaboration	243	Tools for Asynchronous and Time-Zone Flexible Collaboration	Prescriptive	Functional
Group Dynamics	Collaboration	244	User-Friendly Scenario Testing and Reflection Tools	Prescriptive	Functional
Group Dynamics	Collaboration	263	Collaborative Tools for Harnessing Public Creativity and Sentiment Analysis	Prescriptive	Functional
Group Dynamics	Collaboration	271	Real-Time Feedback Mechanisms for Children in DPD	Prescriptive	Functional
Group Dynamics	Collaboration	273	Module-Specific Feedback Collection Tools	Prescriptive	Functional
Group Dynamics	Collaboration	274	In-Situ Feedback Tools for Real-Time User Input	Prescriptive	Functional
Group Dynamics	Communication	27	Enhanced Non-Verbal Communication Tools	Latent	Functional
Group Dynamics	Communication	28	User-Friendly and Inclusive Digital Communication Interfaces	Latent	Non-Functional
Group Dynamics	Communication	29	Effective Communication Strategies	Latent	Non-Functional
Group Dynamics	Communication	31	Digital Communication Tools for e-Planning	Latent	Functional
Group Dynamics	Communication	32	Volunteer Communication Support Tools	Latent	Functional
Group Dynamics	Communication	33	Enhanced Communication Tools for Distributed Teams	Latent	Functional
Group Dynamics	Communication	220	Multimodal Communication Tools	Inferred	Functional
Group Dynamics	Communication	245	Multi-Function Communication Platforms	Prescriptive	Functional
Group Dynamics	Communication	246	Advanced Communication Mechanisms	Prescriptive	Functional
Group Dynamics	Communication	252	Non-Verbal and Visual Communication Tools	Prescriptive	Functional
Group Dynamics	Communication	260	Synchronous and Asynchronous Communication Modes	Prescriptive	Functional

Group Dynamics	Communication	261	Real-Time Communication Tools for Volunteers	Prescriptive	Functional
Group Dynamics	Mitigating Conflict	81	Goal Alignment for Diverse Stakeholders	Latent	Functional
Group Dynamics	Mitigating Conflict	82	Conflict Resolution Tools for Diverse Stakeholder Engagement	Latent	Functional
Group Dynamics	Mitigating Conflict	83	Bridging Tools for Knowledge Integration	Latent	Functional
Group Dynamics	Mitigating Conflict	84	Tools for Managing Stakeholder Conflict and Knowledge Integration	Latent	Functional
Group Dynamics	Mitigating Conflict	85	Flexible Systems for Structuring Uncertain and Complex Problems	Latent	Functional
Group Dynamics	Mitigating Conflict	224	Constructive Discourse Facilitation in Large-Scale Public Engagement	Inferred	Functional
Group Dynamics	Mitigating Conflict	262	Moderation and Translation Tools for Co-Design	Prescriptive	Functional
Group Dynamics	Bridging Diversity	13	Knowledge Dissemination for Diverse Stakeholder Groups	Latent	Functional
Group Dynamics	Bridging Diversity	14	Tools for Bridging Scientific and Local Perspectives	Latent	Functional
Group Dynamics	Bridging Diversity	15	Bridging Professional-Citizen Communication Gap	Latent	Functional
Group Dynamics	Bridging Diversity	16	Bridging Communication Gaps Among Stakeholders	Latent	Functional
Group Dynamics	Bridging Diversity	88	Culturally Sensitive Digital Participation Tools	Latent	Functional
Group Dynamics	Bridging Diversity	89	Culturally Adaptable DPD Platforms	Latent	Functional
Group Dynamics	Bridging Diversity	109	Platforms for Global and Inclusive Participation	Latent	Functional
Group Dynamics	Bridging Diversity	110	Digital Spaces for Multicultural Participation	Latent	Functional
Group Dynamics	Bridging Diversity	111	Communication Tools for Diverse Stakeholder Integration	Latent	Functional
Group Dynamics	Bridging Diversity	161	Facilitation of Knowledge Transfer Between Youth and Decision-Makers	Articulated	Functional
Group Dynamics	Bridging Diversity	185	Broadened Participant Representation Tools	Articulated	Functional
Group Dynamics	Bridging Diversity	186	Intergenerational Collaboration Tools	Articulated	Functional
Group Dynamics	Bridging Diversity	227	Overcoming Language Barriers in International Citizen Science	Inferred	Functional
Group Dynamics	Bridging Diversity	241	Adaptive Tools for Mixed-Ability Participation	Prescriptive	Functional
Group Dynamics	Bridging Diversity	272	Multi-Language Translation Tools for Cross-Cultural PD	Prescriptive	Functional
Group Dynamics	Understanding Sentiments	113	Tools for Monitoring Emotional Dynamics	Latent	Functional
Group Dynamics	Understanding Sentiments	116	Non-Verbal Cue Recognition in Digital Platforms	Latent	Functional
Group Dynamics	Understanding Sentiments	118	Real-Time Engagement and Emotional Cues	Latent	Functional
Group Dynamics	Understanding Sentiments	119	Tools to Mitigate Interpersonal Participation Barriers	Latent	Functional

Group Dynamics	Understanding Sentiments	120	Tools for Experience Exploration	Latent	Functional
Group Dynamics	Understanding Sentiments	286	Sentiment Analysis and Data Integration in Co-Design	Prescriptive	Functional
Group Dynamics	Understanding Sentiments	287	Advanced Sentiment Analysis and Summarization Tools	Prescriptive	Functional
Group Dynamics	Social dynamic	124	Digital Tools for Collaboration and Team Building	Latent	Functional
Group Dynamics	Social dynamic	125	Research-Informed Design for Interactive Engagement	Latent	Non-Functional
Group Dynamics	Social dynamic	126	Facilitation of Informal and Nonverbal Interactions in Virtual Spaces	Latent	Functional
Group Dynamics	Social dynamic	127	Remote Adaptations for Socio-Emotional Interaction	Latent	Functional
Group Dynamics	Social dynamic	128	Tools for Fostering Connectedness in DPD	Latent	Functional
Group Dynamics	Social dynamic	129	Careful Management of Heterogenous Co-Creators	Latent	Non-Functional
Group Dynamics	Social dynamic	130	Rich Interaction Platforms for Enhanced Empathy	Latent	Functional
Group Dynamics	Social dynamic	131	Enhanced Utilization of Networked Structures for Co-Creation	Latent	Functional
Group Dynamics	Social dynamic	132	Technologies for Facilitating Bonding and Deepening Dialogue	Latent	Functional
Group Dynamics	Social dynamic	133	Audio-Visual Tools for Creating Inclusive Environments	Latent	Functional
Group Dynamics	Social dynamic	137	Empathy-Driven Visual Technologies for Enhanced Awareness	Latent	Functional
Group Dynamics	Social dynamic	152	Tools to Mitigate Team Dynamics Challenges in Collaborative Work	Latent	Functional
Group Dynamics	Social dynamic	218	Empathy-Generating Tools for Conflict Mitigation	Articulated	Functional
Group Dynamics	Social dynamic	240	Counteracting Hate Speech	Inferred	Functional
Group Dynamics	Social dynamic	322	Social Recommendation for Topic Highlighting and Collaboration	Prescriptive	Functional
Group Dynamics	Social dynamic	323	Integration of Discussion and Decision Spaces	Prescriptive	Functional
Ensuring Integrity	Accessibility	1	Increasing User Participation	Latent	Non-Functional
Ensuring Integrity	Accessibility	2	Equitable Access to Technology for DPD	Latent	Non-Functional
Ensuring Integrity	Accessibility	3	Accessible Participation Tools	Latent	Non-Functional
Ensuring Integrity	Accessibility	4	Integration of Group-Specific Requirements in Digital Tools	Latent	Functional
Ensuring Integrity	Accessibility	5	Integrative Approach for Public Participation	Latent	Functional
Ensuring Integrity	Accessibility	6	Addressing the Digital Divide for Equal Participation	Latent	Non-Functional
Ensuring Integrity	Accessibility	7	Inclusive Digital Access Tools	Latent	Functional
Ensuring Integrity	Accessibility	8	Accessible Tools for Efficient Ideation In Online Setting	Latent	Functional

Ensuring Integrity	Accessibility	9	Remote Alternatives for Inclusivity in PD	Latent	Functional
Ensuring Integrity	Accessibility	10	Inclusive Digital Access	Latent	Non-Functional
Ensuring Integrity	Accessibility	11	Accessible Participation Tools for Diverse Users	Latent	Functional
Ensuring Integrity	Accessibility	12	Inclusive Digital Learning Co-Design Tools for Children	Latent	Functional
Ensuring Integrity	Accessibility	167	Affordable and Accessible Technology Solutions	Articulated	Non-Functional
Ensuring Integrity	Accessibility	168	Broad Participation in Data Collection Processes	Articulated	Functional
Ensuring Integrity	Accessibility	169	User-Friendly Interface for Diverse Participants	Articulated	Non-Functional
Ensuring Integrity	Accessibility	170	Adaptive and Environment-Specific Solutions	Articulated	Non-Functional
Ensuring Integrity	Accessibility	171	Inclusive ICT Solutions for Public Spaces	Articulated	Non-Functional
Ensuring Integrity	Accessibility	172	Child-Inclusive Design Tools	Articulated	Non-Functional
Ensuring Integrity	Accessibility	247	Co-Design Tools for ASD-Focused Participatory Design	Prescriptive	Functional
Ensuring Integrity	Accessibility	248	Child-Friendly Digital Tools for Remote Participation	Prescriptive	Functional
Ensuring Integrity	Accessibility	249	Child-Centric PD Collaboration Tools	Prescriptive	Functional
Ensuring Integrity	Accessibility	250	Flexible Scheduling and Location Tools for Participation	Prescriptive	Functional
Ensuring Integrity	Accessibility	251	Improved Age Group Adaptability in Platform Design	Prescriptive	Non-Functional
Ensuring Integrity	Accessibility	258	User-Friendly Data Collection Tools for Broad Participation	Prescriptive	Functional
Ensuring Integrity	Accessibility	259	Child-Friendly Distributed Co-Design Tools	Prescriptive	Functional
Ensuring Integrity	Engagement	71	User Engagement Features for Sustained Participation	Latent	Functional
Ensuring Integrity	Engagement	72	Tools for Enhanced Online Engagement Monitoring	Latent	Functional
Ensuring Integrity	Engagement	73	Improved Engagement and Quality Control in Digital Collaboration	Latent	Non-Functional
Ensuring Integrity	Engagement	74	Comprehensive Platform for End-to-End Civic Engagement	Latent	Functional
Ensuring Integrity	Engagement	75	Accessible and Flexible Civic Engagement Tools	Latent	Functional
Ensuring Integrity	Engagement	76	Customizable Engagement Frameworks	Latent	Functional
Ensuring Integrity	Engagement	77	Interactive Digital Platforms for Active Citizen Involvement	Latent	Functional
Ensuring Integrity	Engagement	78	Comprehensive Technological Support for Stakeholder Engagement	Latent	Functional
Ensuring Integrity	Engagement	79	Tools for Enhanced Engagement and Inclusion in Digital Participatory Design	Latent	Functional
Ensuring Integrity	Engagement	80	Tools for Active Stakeholder Engagement	Latent	Functional

Ensuring Integrity	Engagement	99	Context-Sensitive Participatory Modeling Frameworks	Latent	Functional
Ensuring Integrity	Engagement	175	Engaging Hard-to-Reach Populations in Urban Planning	Articulated	Functional
Ensuring Integrity	Engagement	176	Ensuring Engagement and Adequate Contributions via Platform Design	Articulated	Functional
Ensuring Integrity	Engagement	177	Enhanced Spatial Quality and Community Participation in Decision-Making	Articulated	Functional
Ensuring Integrity	Engagement	178	Engagement and Data Management Tools	Articulated	Functional
Ensuring Integrity	Engagement	179	Mobile Technologies for Co-Creators Engagement in Co-Creation	Articulated	Functional
Ensuring Integrity	Engagement	225	Active User Engagement with Interactive Design Features	Inferred	Non-Functional
Ensuring Integrity	Engagement	266	Addressing Engagement, Data Management, and Internet Equity	Prescriptive	Non-Functional
Ensuring Integrity	Engagement	267	Continuous Engagement via e-Planning Platforms	Prescriptive	Functional
Ensuring Integrity	Engagement	268	User-Friendly Interaction Interfaces To Facilitate Participation	Prescriptive	Non-Functional
Ensuring Integrity	Engagement	269	Cost-Effective Engagement and Visualization Tools	Prescriptive	Functional
Ensuring Integrity	Representativity	100	Transformative AI Support for Inclusive Planning	Latent	Functional
Ensuring Integrity	Representativity	103	Enhancing Collective Intelligence through Digital Tools	Latent	Functional
Ensuring Integrity	Representativity	104	Mitigating Risks of Manipulation, Centralized Power, and Privacy Breach	Latent	Non-Functional
Ensuring Integrity	Representativity	105	Tools to Ensure Representative Participation in Co-Design	Latent	Functional
Ensuring Integrity	Representativity	106	Tools for Minimizing Bias in Citizen Science	Latent	Functional
Ensuring Integrity	Representativity	107	Overcome Selection Bias Participants Group	Latent	Non-Functional
Ensuring Integrity	Representativity	108	Addressing Inclusivity and Equity Challenges	Latent	Non-Functional
Ensuring Integrity	Representativity	112	Tools for Democratizing Participation and Redistributing Power	Latent	Functional
Ensuring Integrity	Representativity	114	Equity-Focused Tools to Prevent Process Domination	Latent	Functional
Ensuring Integrity	Representativity	115	Tools for Mitigating Power Asymmetries in Civic Design	Latent	Functional
Ensuring Integrity	Representativity	195	Bias Mitigation in Technology Design	Articulated	Non-Functional
Ensuring Integrity	Representativity	197	Mitigating Contributor Bias in Participation	Articulated	Non-Functional
Ensuring Integrity	Representativity	204	Addressing Contribution Centralization	Articulated	Non-Functional
Ensuring Integrity	Representativity	205	Tool Mix for Reducing Selection Bias	Articulated	Non-Functional
Ensuring Integrity	Representativity	206	Secure and Equitable Collaborative Workflows	Articulated	Non-Functional
Ensuring Integrity	Representativity	278	AI-Simulating Stakeholder Perspectives	Prescriptive	Functional

Ensuring Integrity	Representativity	279	Reducing Self-Selection Bias	Prescriptive	Non-Functional
Ensuring Integrity	Representativity	280	Multimodal Participant Selection	Prescriptive	Functional
Ensuring Integrity	Security and Privacy	117	Privacy-Enhanced Tools for Data Security and Transparency	Latent	Functional
Ensuring Integrity	Security and Privacy	122	Privacy and Security Solutions for Mobile Co-Creation	Latent	Functional
Ensuring Integrity	Security and Privacy	213	Security Assurance in Collaborative Processes	Articulated	Non-Functional
Ensuring Integrity	Security and Privacy	288	Enhanced Security Features for CDMP	Prescriptive	Non-Functional
Ensuring Integrity	Security and Privacy	289	Secure Communication Platforms for Artifact Sharing	Prescriptive	Non-Functional
Ensuring Integrity	Security and Privacy	290	Traceability Features with Privacy Safeguards	Prescriptive	Functional
Ensuring Integrity	Security and Privacy	291	Trusted Data Storage and Computation through DLT	Prescriptive	Functional
Ensuring Integrity	Security and Privacy	292	Advanced Encryption for Privacy and Anonymity	Prescriptive	Non-Functional
Ensuring Integrity	Security and Privacy	293	Data Security and Usability	Prescriptive	Non-Functional
Ensuring Integrity	Security and Privacy	294	Secure and Transparent Citizen Engagement Tools	Prescriptive	Non-Functional
Ensuring Integrity	Accountability and Transparency	144	Enhanced Transparency, Accountability, and Security in Digital Participation	Latent	Non-Functional
Ensuring Integrity	Accountability and Transparency	145	Transparent Communication in Public Planning	Latent	Functional
Ensuring Integrity	Accountability and Transparency	146	Digital Tools for Transparency and Collaboration	Latent	Functional
Ensuring Integrity	Accountability and Transparency	147	Transparency and Legitimacy through Information Sharing	Latent	Non-Functional
Ensuring Integrity	Accountability and Transparency	153	Enhancing Transparency and Accountability	Latent	Non-Functional
Ensuring Integrity	Accountability and Transparency	154	Transparency and Trust-Building Mechanisms	Latent	Functional
Ensuring Integrity	Accountability and Transparency	155	Transparent and Fair Decision-Making Tools	Latent	Functional
Ensuring Integrity	Accountability and Transparency	156	Ensuring Transparency and Inclusion	Latent	Non-Functional
Ensuring Integrity	Accountability and Transparency	157	Solutions Transparency	Latent	Non-Functional
Ensuring Integrity	Accountability and Transparency	158	Transparent Activity Tracking and Leadership Tools	Latent	Functional

Ensuring Integrity	Accountability and Transparency	159	IT Tools for Enhancing Accountability and Stakeholder Relations	Latent	Functional
Ensuring Integrity	Accountability and Transparency	160	Data Usage Transparency Tools	Latent	Functional
Ensuring Integrity	Accountability and Transparency	217	Trust and Transparency Enhancement	Articulated	Non-Functional
Ensuring Integrity	Accountability and Transparency	239	Institutional Memory Tools for Transparent Participant History	Inferred	Functional
Ensuring Integrity	Accountability and Transparency	327	Scalable Blockchain-Based Accountability Tools for E-Government	Prescriptive	Functional
Ensuring Integrity	Accountability and Transparency	328	Secure Contribution Tracking and Safeguarding	Prescriptive	Functional
Enabling Methodology	Decision-Making	38	Flexible Voting Methods for Context-Specific Decision-Making	Latent	Functional
Enabling Methodology	Decision-Making	39	Accessible Decision-Making Models for Public Engagement	Latent	Functional
Enabling Methodology	Decision-Making	40	Enhanced Digital Decision-Making Mechanisms	Latent	Functional
Enabling Methodology	Decision-Making	41	Accessible and Practical Decision Support Systems	Latent	Functional
Enabling Methodology	Decision-Making	42	Empowerment Tools for Shared Decision-Making	Latent	Functional
Enabling Methodology	Decision-Making	43	Inclusive Decision-Making Processes	Latent	Functional
Enabling Methodology	Decision-Making	183	AI Integration for Enhanced Decision-Making	Articulated	Functional
Enabling Methodology	Decision-Making	188	Enhanced Participation In Decision Making Process	Articulated	Functional
Enabling Methodology	Decision-Making	189	Advanced Decision Support System	Articulated	Functional
Enabling Methodology	Decision-Making	297	Decision-Support Tools for Civic Engagement	Prescriptive	Functional
Enabling Methodology	Decision-Making	298	Reliable Digital Decision Support Systems	Prescriptive	Non-Functional
Enabling Methodology	Decision-Making	299	Automated Deliberative Process Monitoring Tools	Prescriptive	Functional
Enabling Methodology	Decision-Making	300	Enhanced Deliberative Features for E-Planning	Prescriptive	Functional
Enabling Methodology	Decision-Making	301	Technological Tools for Diverse Decision-Making	Prescriptive	Functional
Enabling Methodology	Distributed Process	44	Distributed Participatory Design Support	Latent	Functional
Enabling Methodology	Distributed Process	45	Support for Flexible Distributed Participation	Latent	Functional
Enabling Methodology	Distributed Process	46	Digital Systems for Public Participation and Education	Latent	Functional
Enabling Methodology	Distributed Process	47	Integration of Remote Methods in PD	Latent	Functional

Enabling Methodology	Distributed Process	48	Simplified Remote Citizen Involvement	Latent	Functional
Enabling Methodology	Distributed Process	49	Frameworks for Organizing Distributed Participatory Design	Latent	Functional
Enabling Methodology	Distributed Process	190	Accessible and Engaging Virtual Participation Platforms	Articulated	Non-Functional
Enabling Methodology	Distributed Process	191	Enhanced Facilitation Tools for Distributed Co-Design	Articulated	Functional
Enabling Methodology	Distributed Process	192	Seamless Distributed Co-Design Tools	Articulated	Functional
Enabling Methodology	Distributed Process	302	Hybrid Participation Tools for Augmenting In-Person Engagement	Prescriptive	Functional
Enabling Methodology	New Methods	50	Advanced Geospatial Tools for Complex Areas	Latent	Functional
Enabling Methodology	New Methods	51	Development of Methods and Digital Tools for Online Workshops	Latent	Functional
Enabling Methodology	New Methods	52	Improved Online Workshops	Latent	Functional
Enabling Methodology	New Methods	53	Expanded E-Participation Frameworks	Latent	Functional
Enabling Methodology	New Methods	54	Adaptive E-Participation Methods	Latent	Functional
Enabling Methodology	New Methods	55	Standardized and Effective E-Participation Techniques	Latent	Functional
Enabling Methodology	New Methods	56	Bridging Theory and Practice through IT	Latent	Functional
Enabling Methodology	New Methods	194	Participatory Mapping Technology	Articulated	Functional
Enabling Methodology	New Methods	198	Integration of Emerging Technologies in DPD Tools	Articulated	Functional
Enabling Methodology	New Methods	199	Expanded Reach for Co-Design Techniques	Articulated	Functional
Enabling Methodology	Evidence-Based Process	57	Accessible Data for Informed Public Opinions	Latent	Non-Functional
Enabling Methodology	Evidence-Based Process	58	Tools for Evidence-Based Communication Among Stakeholders	Latent	Functional
Enabling Methodology	Evidence-Based Process	59	Knowledge-to-Policy Integration Tools	Latent	Functional
Enabling Methodology	Evidence-Based Process	232	Counteracting Misinformation	Inferred	Functional
Enabling Methodology	Evidence-Based Process	306	Accessible Scientific Evidence through AI	Prescriptive	Functional
Enabling Methodology	Evidence-Based Process	307	Accurate Data Collection and Analysis for Decision-Making	Prescriptive	Functional
Enabling Methodology	Evidence-Based Process	308	Reliable and Discrete Information	Prescriptive	Non-Functional
Enabling Methodology	Evidence-Based Process	309	NLP for Policy Narrative Analysis	Prescriptive	Functional
Enabling Methodology	Large Scale Process	60	Digital Tools for Large-Scale Creative Public Participation	Latent	Functional
Enabling Methodology	Large Scale Process	61	Scalable Co-Design Practices for Broad Public Engagement	Latent	Non-Functional
Enabling Methodology	Large Scale Process	68	Scalable Digital Participation Channels	Latent	Non-Functional

Enabling Methodology	Large Scale Process	69	Large-Scale Participant Inclusion	Latent	Non-Functional
Enabling Methodology	Large Scale Process	70	Structured Approaches for Asynchronous Participation in Large Distributed Projects	Latent	Functional
Enabling Methodology	Large Scale Process	90	Scalable Digital Participation Solutions	Latent	Non-Functional
Enabling Methodology	Large Scale Process	203	Scalable Co-Design Tools for Large-Scale Public Engagement	Articulated	Non-Functional
Enabling Methodology	Large Scale Process	317	Feedback Management and Moderation Tools for Large-Scale Participation	Prescriptive	Functional
Enabling Methodology	Large Scale Process	318	Ubiquitous Tools for Large-Scale Data Collection	Prescriptive	Functional
Enabling Methodology	Large Scale Process	319	High-Volume Online Participation Tools	Prescriptive	Functional
Enabling Methodology	Specific Tools	91	General-Purpose Participatory Planning System	Latent	Functional
Enabling Methodology	Specific Tools	92	Integrated System for Public Participation	Latent	Functional
Enabling Methodology	Specific Tools	93	Ubiquitous Digital Tools for Citizen Science Integration	Latent	Functional
Enabling Methodology	Specific Tools	94	Holistic Collaborative Platform for Co-Production	Latent	Functional
Enabling Methodology	Specific Tools	95	Comprehensive Digital Toolkits for Co-Design	Latent	Functional
Enabling Methodology	Specific Tools	96	Increased Empirical Applications of Co-Creation Tools	Latent	Non-Functional
Enabling Methodology	Specific Tools	97	Development of Customized Native Technologies	Latent	Functional
Enabling Methodology	Specific Tools	98	Adaptive Systems for Dynamic User Requirements	Latent	Functional
Enabling Methodology	Specific Tools	207	Systems for Dedicated Participatory Design Support	Articulated	Functional
Enabling Methodology	Specific Tools	208	Digital System for Decision-Making Support in Urban Design	Articulated	Functional
Enabling Methodology	Specific Tools	209	Integrated Digital System for Urban Design Communication and Collaboration	Articulated	Functional
Enabling Methodology	Specific Tools	210	Advanced Digital Tools for Urban Stakeholder Participation	Articulated	Functional
Enabling Methodology	Specific Tools	211	Open-Ended and Tailorable Software Environments	Articulated	Functional
Enabling Methodology	Specific Tools	234	Integrated and Usable Solutions for Sustainable Planning	Inferred	Functional
Enabling Methodology	Specific Tools	326	Stage-Specific Digital Tools for Public Participation	Prescriptive	Functional
Process Management	Data Management	30	Enhanced Data Collection and Analysis Capabilities	Latent	Functional
Process Management	Data Management	34	Data Aggregation and Analysis Tools for Civic Engagement	Latent	Functional
Process Management	Data Management	35	Data Collection and Knowledge Transfer Tools	Latent	Functional
Process Management	Data Management	36	Digitized Data Aggregation and Analysis Tools	Latent	Functional
Process Management	Data Management	37	AI-Enhanced Crowdsourced Data Science Tools	Latent	Functional

Process Management	Data Management	102	Digital Support for Idea Analysis and Organization	Latent	Functional
Process Management	Data Management	166	User-Centered Geospatial Data Collection Tools	Articulated	Functional
Process Management	Data Management	173	Data Management Tools	Articulated	Functional
Process Management	Data Management	182	Enhanced Integration of GIS Tools for Urban Planning	Articulated	Functional
Process Management	Data Management	221	Data Management and Filtering Tools for Crowdsourced Data	Inferred	Functional
Process Management	Data Management	222	High-Accuracy Data Collection and Validation Tools	Inferred	Functional
Process Management	Data Management	223	Standardized and Integrated Data Platforms	Inferred	Functional
Process Management	Data Management	226	E-Planning GIS for Multi-Dimensional Impact Assessment	Inferred	Functional
Process Management	Data Management	228	Automated Idea Organization in Participatory Design	Inferred	Functional
Process Management	Data Management	253	Advanced Data Processing Capabilities	Prescriptive	Functional
Process Management	Data Management	254	Enhanced Data Analysis Technology in Co-Creation	Prescriptive	Functional
Process Management	Data Management	255	Comprehensive Data Management and Engagement Tools	Prescriptive	Functional
Process Management	Data Management	256	Real-Time Data Collection Capabilities	Prescriptive	Functional
Process Management	Data Management	257	Multi-Format Data Collection Tools	Prescriptive	Functional
Process Management	Data Management	264	Data Analytics for Participatory Platforms	Prescriptive	Functional
Process Management	Data Management	265	NLP for Data Synthesis and Categorization in Planning	Prescriptive	Functional
Process Management	Data Management	270	GIS Integration for Urban Impact Assessment	Prescriptive	Functional
Process Management	Data Management	277	Integrated Web-GIS Tools	Prescriptive	Functional
Process Management	Information and Knowledge	121	Tools for Evaluating Unstructured Feedback	Latent	Functional
Process Management	Information and Knowledge	212	Developer's Blog for Ongoing Project Updates	Articulated	Functional
Process Management	Information and Knowledge	231	Tools for Summarizing and Structuring Discussion Threads	Inferred	Functional
Process Management	Information and Knowledge	281	Improved Information Management Systems for Stakeholder Access	Prescriptive	Functional
Process Management	Information and Knowledge	282	NLP-Based Relevance Scoring for Contribution Filtering	Prescriptive	Functional
Process Management	Information and Knowledge	283	Information Management Tools for Co-Creation	Prescriptive	Functional
Process Management	Information and Knowledge	284	Semantization Tools for Knowledge Structuring	Prescriptive	Functional
Process Management	Information and Knowledge	285	Digital Platform for Co-Design Knowledge and Guidance	Prescriptive	Functional
Process Management	Process Optimisation	123	Resource-Efficient Digital Facilitation Tools for Co-Design	Latent	Functional

Process Management	Process Optimisation	134	Flexible Participation Tools for Improved Engagement	Latent	Functional
Process Management	Process Optimisation	135	Decision Chain Acceleration Tools	Latent	Functional
Process Management	Process Optimisation	136	NLP Tools for Ideation and Evaluation	Latent	Functional
Process Management	Process Optimisation	138	Overcoming Geographic and Financial Barriers in PD	Latent	Non-Functional
Process Management	Process Optimisation	139	Improving Implementation Processes	Latent	Non-Functional
Process Management	Process Optimisation	140	Enhancing Capacity for Proposal Processing	Latent	Non-Functional
Process Management	Process Optimisation	141	Efficient Co-Creation Processes Upholding Democratic Principles	Latent	Non-Functional
Process Management	Process Optimisation	214	Digital System for Simplified Public Participation	Articulated	Functional
Process Management	Process Optimisation	215	Multi-Tool Resource Efficiency	Articulated	Non-Functional
Process Management	Process Optimisation	216	Outcome Assessment for Improvement	Articulated	Functional
Process Management	Process Optimisation	236	Reduction of Tedious Steps in Participatory Processes	Inferred	Non-Functional
Process Management	Process Optimisation	237	Time-Efficient and Cost-Effective Digital Collaboration Tools	Inferred	Non-Functional
Process Management	Process Optimisation	275	AI-Based Tools for Input and Feedback Evaluation	Prescriptive	Functional
Process Management	Process Optimisation	276	Automatic Title Generation and Summarization Tools	Prescriptive	Functional
Process Management	Organisation	142	Technology-Enhanced Participant Recruitment and Retention	Latent	Functional
Process Management	Organisation	143	Cross-Organizational Coordination Tools	Latent	Functional
Process Management	Organisation	148	Real-Time Interactive AI	Latent	Functional
Process Management	Organisation	149	Integrated Management Tools for E-Participation Components	Latent	Functional
Process Management	Organisation	150	Comprehensive Process Representation Tools	Latent	Functional
Process Management	Organisation	151	ML and AI-Enhanced Facilitation Tools	Latent	Functional
Process Management	Organisation	238	Feedback Capture Mechanisms for In-Person Engagement	Inferred	Functional
Process Management	Organisation	320	AI Tools for Facilitation and Analysis in DPD	Prescriptive	Functional
Process Management	Organisation	321	Targeted Digital Platforms for Co-Design Tasks	Prescriptive	Functional
Process Management	Organisation	324	Integrated Workspace for Information Sharing and Task Management	Prescriptive	Functional
Process Management	Organisation	325	Effective Contribution Management	Prescriptive	Functional
